# The barriers and enablers to accessing sexual health and sexual well-being services for midlife women (aged 40–65 years) in high-income countries: A mixed-methods systematic review

**DOI:** 10.1177/17455057241277723

**Published:** 2024-09-21

**Authors:** Kiersten Simmons, Carrie Llewellyn, Stephen Bremner, Yvonne Gilleece, Claire Norcross, Collins Iwuji

**Affiliations:** 1 Brighton and Sussex Medical School, and University Hospitals Sussex NHS Foundation Trust, Brighton, UK; 2 Department of Primary Care and Public Health, Brighton and Sussex Medical School, Brighton, UK; 3 London School of Hygiene and Tropical Medicine, London, UK; 4 Global Health and Infection Department, Brighton and Sussex Medical School and University Hospitals Sussex NHS Foundation Trust, Brighton, UK; 5 Africa Health Institute, KwaZulu-Natal, South Africa

**Keywords:** access, women, middle age, sexual health, sexual well-being

## Abstract

Midlife, beginning at 40 years and extending to 65 years, a range that encompasses the late reproductive to late menopausal stages, is a unique time in women’s lives, when hormonal and physical changes are often accompanied by psychological and social evolution. Access to sexual health and sexual well-being (SHSW) services, which include the prevention and management of sexually transmitted infections, contraception and the support of sexual function, pleasure and safety, is important for the health of midlife women, their relationships and community cohesion. The objective was to use the socio-ecological model to synthesise the barriers and enablers to SHSW services for midlife women in high-income countries. A systematic review of the enablers and barriers to women (including trans-gender and non-binary people) aged 40–65 years accessing SHSW services in high-income countries was undertaken. Four databases (PubMed, PsycINFO, Web of Science and Google Scholar) were searched for peer-reviewed publications. Findings were thematically extracted and reported in a narrative synthesis. Eighty-one studies were included; a minority specifically set out to study SHSW care for midlife women. The key barriers that emerged were the intersecting disadvantage of under-served groups, poor knowledge, about SHSW, and SHSW services, among women and their healthcare professionals (HCPs), and the over-arching effect of stigma, social connections and psychological factors on access to care. Enablers included intergenerational learning, interdisciplinary and one-stop women-only services, integration of SHSW into other services, peer support programmes, representation of minoritised midlife women working in SHSW, local and free facilities and financial incentives to access services for under-served groups. Efforts are needed to enhance education about SHSW and related services among midlife women and their healthcare providers. This increased education should be leveraged to improve research, public health messaging, interventions, policy development and access to comprehensive services, especially for midlife women from underserved groups.

## Plain language summary

Sexual health and sexual wellbeing services for midlife women in high income countries

Midlife, beginning at 40 years and extending to 65 years, a range that encompasses the late reproductive to late menopausal stages, is a unique time in women’s lives. Access to Sexual Health and Sexual Wellbeing (SHSW) services, which include the prevention and management of sexually transmitted infections, contraception and the support of sexual function, pleasure and safety, is important for the health of midlife women, their relationships and community cohesion. The objective of this systematic review was to use the socio-ecological model to synthesise the barriers and enablers to SHSW services for midlife women in high income countries. Eighty-one studies were included; a minority specifically set out to study SHSW care for midlife women. The key barriers that emerged were the intersecting disadvantage of under-served groups, poor knowledge, about SHSW, and SHSW services, among women and their HealthCare Professionals (HCPs), and the over-arching effect of stigma, social connections, and psychological factors on access to care. Enablers included intergenerational learning, interdisciplinary and one-stop women-only services, integration of SHSW into other services, peer support programmes, representation of minoritised midlife women working in SHSW, local and free facilities, and financial incentives for under-served groups to access services. The appetite for education about SHSW and SHSW services among midlife women and their HCPs should be capitalised upon, and utilised to improve research, public health messaging, interventions and access to holistic services, particularly for midlife women from under-served groups.

## Introduction

The publication of the UK government’s first Women’s Health Strategy in 2022^1^ coincided with the welcome and long overdue global recognition of the necessity for more multi-disciplinary research in both women’s^2–5^ and older people’s sexual health and sexual well-being (SHSW).^6–8^ Public health approaches to sexuality focus on adverse biomedical outcomes,^9^ despite the growing evidence for the importance of a holistic approach when evaluating sexual health,^8,9^ and the inclusion of positive sexuality and sexual experiences in the World Health Organization’s definition of sexual health.^10^ In this review, we have followed Mitchell’s framework, which separates SHSW into two separate, inter-linked foci of public health enquiry.^11^ Sexual health is defined as encompassing the prevention and management of sexually transmitted infections (STIs) including HIV, sexual violence prevention, support of sexual function, desire and arousal and fertility management. Sexual well-being is a multi-dimensional concept,^12,13^ which comprises sexual safety and security, sexual respect, sexual self-esteem, resilience in relation to sexual experience, forgiveness of past sexual experience, comfort with sexuality and self-determination in one’s sexual life.^11^ SHSW services are often combined and offered in primary care, secondary care (sexual health and gynaecology services), health services which look after physical and mental health conditions interlinked with SHSW, and community and charitable services. Midlife is defined as beginning at 40 years and extending to 65 years,^14^ a time when hormonal, physical and psychological changes in the lives of women^15^ are often accompanied by evolution in their relationships,^16,17^ and social developments.^18^ This stage can be a positive transition point for women,^19^ empowering them to determine how they envision spending the latter half of their years, and can result in varied sexual experiences.^20^

Midlife women are a growing population, often responsible for being both primary caretakes of children and elders, and major economic contributors.^21,22^ Increasing societal demands impact on their sexual experiences.^23^ Cultural shifts in relationship patterns,^24^ and population changes due to the stretching of mid years,^25^ mean that SHSW services are an increasingly important part of the lives of midlife women, integral to the physical and mental health of many.^26,27^ Intimate partner violence and sexual assault are major public health issues, which have long-term effects on the health and functioning of many midlife women.^28^ Prompt, effective diagnosis and treatment of STIs and gynaecological diseases, and the provision to support midlife women in having enjoyable, safe sex, has wide-ranging benefits to individuals, partnerships^29^ and communities.^30^ Indeed, the provision of sexual and reproductive health services is related to multiple human rights and should be available in adequate numbers, physically and economically accessible without discrimination and of good quality for everybody, including all midlife women.^31^ Studies are often framed in a male, hetero-normative^32^ perspective and the heterogeneous needs of midlife women are sometimes stereotyped by ageist and sexist misconceptions, or forgotten,^24^ squeezed between the higher prevalence of STIs in adolescents and the increasingly apparent high prevalence of sexual dysfunction in older adults.^33^

General health issues, such as diabetes, cardiovascular disease and arthritis, begin to emerge during the latter half of midlife and operate both to impact on sexual experience, with implications for the need for help-seeking, and should also provide opportunities for issues relating to sexual matters to be raised in routine healthcare.^8^ However, many healthcare professionals (HCPs) are reserved about, and ill equipped to discuss SHSW with midlife women.^5,6,15,32,34–37^ Stigma and negative attitudes about women’s sexuality, and sexuality at older ages, may inhibit discussion with HCPs.^38^ Embarrassment may inhibit women from accessing help when symptomatic,^15^ and SHSW services are not designed in a way that encourage midlife adults to seek help.^5,6,15,32,34–37^ Health policies about ageing often omit sexuality, further contributing to the lack of research and services available.^39^ Technology has not been sufficiently harnessed; the only evidence available for improving access to SHSW services for midlife women lies in the menopause field, but many menopause apps lack a credible evidence base.^40^

In addition to the health inequalities related to identifying as female gender,^41^ including the diminishing healthy life expectancy despite a longer life expectancy of women,^42^ and the historical default of medical research being carried out on men,^43^ there are also significant disparities in access to, and engagement with, healthcare services among women, largely due to the social determinants of health.^44^ Within globally privileged settings, there is already a high disparity in access to services. The review was therefore restricted to high-income countries to improve the specificity of findings. It has enabled us to disentangle the impact of different strands of marginalisation within the context of affluent economies, which have separate challenges from low- and middle-economic locales. The World Bank’s definition of high-income countries was employed; high-income countries had a gross national income per capita of US$13,845 or more in 2022, calculated using the Atlas method.^45^

This review evaluated the research that identified the enablers and barriers to midlife women’s access to SHSW services and established whether any groups of midlife women were particularly disadvantaged. It thereby delineated the key foci for policy and strategy change to improve quality and equity of SHSW care for midlife women in high-economic countries. The socio-ecological model (SEM) recognises the multiple and dynamic factors that can affect the barriers and enablers to accessing healthcare by considering the complex interplay between individual, interpersonal, organisational, community and public policy factors.^46^ These factors affect SHSW decision-making for an individual and can both enable and inhibit healthy sexual behaviours.^47^ The SEM approach has been successfully employed to interrogate many aspects of SHSW services for different populations, for example SHSW services for female sex workers,^48^ adolescents^49,50^ and migrant Asian women,^51^ and has enabled a holistic consideration of the different SHSW needs of midlife women within the complex context of the societies in which they live.

### Objectives

The objectives of this review were to identify the barriers and enablers to SHSW services for midlife women in high-income countries^45^ using the SEM,^52^ and to identify the groups of midlife women in high-income countries who found accessing these services particularly challenging.

## Method

**Figure 1.**
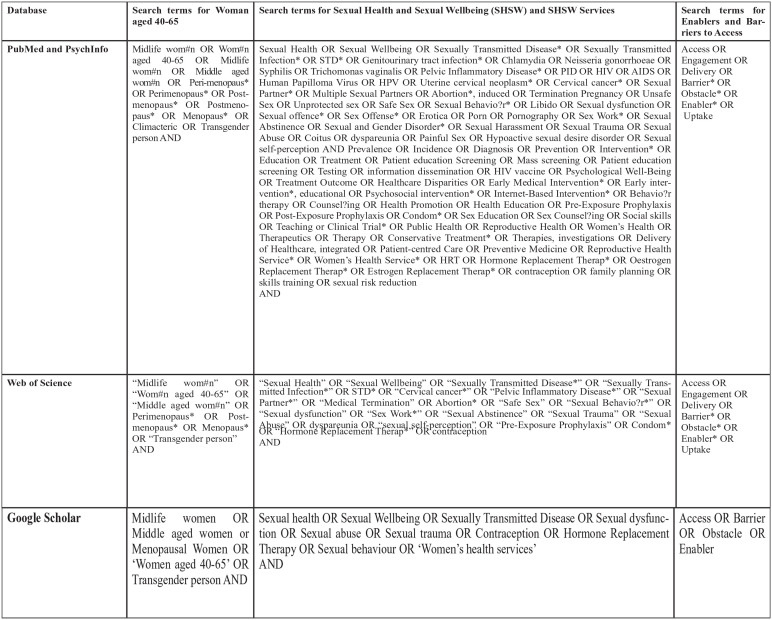
PRISMA flow diagram. The barriers and enablers to accessing sexual health and well-being services for midlife women (aged 40–65 years) in high-income countries: A mixed-methods systematic review. PRISMA, Preferred Reporting Items for Systematic Reviews and Meta-Analysis. Source: Page MJ, McKenzie JE, Bossuyt PM, et al. The PRISMA 2020 statement: an updated guideline for reporting systematic reviews. *BMJ* 2021; 372: n71. Doi: 10.1136/bmj.n71. For more information, visit: http://www.prisma-statement.org/

Reporting of the review followed the Preferred Reporting Items for Systematic Reviews and Meta-Analysis (PRISMA) recommendations (Figure 1). The protocol was Registered with the PROSPERO database on 8 June 2023, registration number: CRD42023433812.

### Eligibility criteria

The inclusion criteria (Table 1) were studies that had been published between November 1993 and November 2023, peer-reviewed, employed any methodology to capture primary data and investigated barriers to, or enablers of, access to SHSW care for midlife women (40–65 years old) who resided in high-income countries,^45^ grouped into intrapersonal, HCP relationship, community and organisation perspectives. Studies on anyone who identified as a woman, gender diverse and non-conformist to either gender were included in this review. Transgender women and non-binary people (TGNB) experience different levels of stigma and discrimination than their cisgender counterparts,^53^ but can share more in common with cisgender women than they do with men who have sex with men,^54^ a group with whom they are often aggregated in SHSW research.^55^ The review states when conclusions were drawn from TGNB-specific studies.

**Table 1.** Inclusion and exclusion criteria for the barriers and enablers to accessing sexual health and sexual well-being services for midlife women (aged 40–65 years) in high-income countries: A mixed-methods systematic review.

Study characteristic	Inclusion criteria	Exclusion criteria
Sample	Studies which were composed of self-identifying women aged 40–65 years (studies which included men and women were included if women aged 40–65 years (or whose mean age was between 40 and 65) represented 50% or more of the study population, or women aged 40–65 were analysed separately). Studies which were composed of HCPs who worked in, or sign-posted midlife women to, SHSW services.	Studies which were composed of less than 50% self-identifying women aged 40–65 (or whose mean age was not between 40 and 65) or studies in which women aged 40–65 were not analysed separately. Studies which were composed of HCPs who did not work with or sign post-midlife women to SHSW services.
Design	Empirical research.	Review/Meta-analysis articles. Theoretical articles. Book chapters. Unpublished manuscripts. Conference abstracts.
Publication	Peer-reviewed. Published between November 1993 and October 2023.	Not peer-reviewed. Published prior to November 1993 or after October 2023.
Language	English.	Any other language.
Duration of follow-up	All.	Not applicable.
Focus	Studies that assessed intra-personal-based factors, for example, age, sexuality, ethnicity, knowledge, psychological factors, other priorities, that may have affected midlife women’s access to SHSW services. Studies which evaluated the impact of interactions with HCPs on access to SHSW services. Studies which investigated the impact of organisational factors (hospital policies) on access to SHSW services. Studies which investigated community factors, for example, social stigma, which impacted on access to SHSW services. Studies which investigated public policy, for example health insurance coverage, guidelines, which impacted on access to SHSW services.	Studies which investigated interventions, for example, treatment for menopause, sexual dysfunction, HIV testing, adherence to HIV pre-exposure prophylaxis, adherence to antiretrovirals, if not directly related to improving access to existing services. Studies which investigated the enablers and barriers to fertility technology techniques. Studies which investigated the enablers and barriers to accessing male-related sexual dysfunction services. Studies which assessed access to gender-affirming therapy for transpeople. Studies which investigated the impact of COVID-19 or other natural disasters on barriers/facilitators to services unless it included barriers/facilitators applicable to ongoing care. Studies with a focus on enablers and barriers to accessing SHSW care for men who have sex with men and non-binary and/ or transpeople unless there was a separate analysis for midlife non-binary and/ or transpeople, or midlife transpeople represented at least 50% of the studied population.

HCP, healthcare professionals; SHSW, sexual health and sexual well-being.

Exclusion criteria (Table 1) comprised studies which investigated fertility technology techniques, as this was felt to widen the scope of the review too much and warrant a separate analysis. Male-related sexual dysfunction services were excluded, as although these would affect some women as partners in a relationship, women would not be the primary focus of the services. Studies addressing enablers and barriers to care for midlife TGNB people were only included in the review if they met the criteria of including at least 50% TGNB people and did not focus solely on gender-affirming therapy, a subject that warrants a separate review. COVID-19 may have had a significant impact on access to SHSW services^48^; studies were only included if enablers and barriers that could be attributed to the COVID-19 pandemic had not yet reverted back to their state prior to the pandemic.

### Search strategy

**Figure 2.**
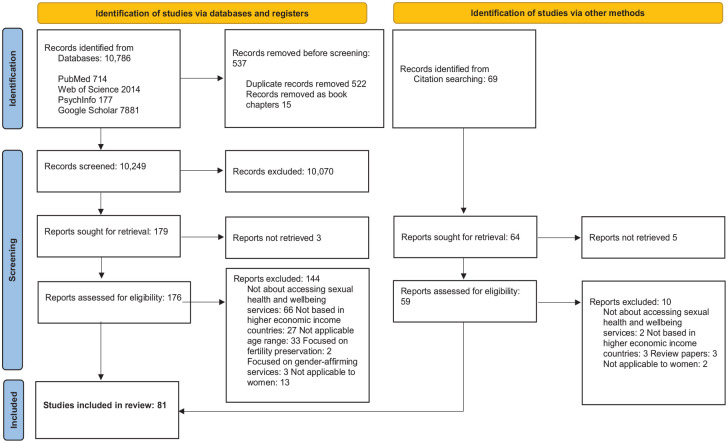
Detailed search strategy criteria for the barriers and enablers to accessing sexual health and well-being services for midlife women (aged 40–65 years) in high-income countries: a mixed-methods systematic review.

The search strategy was devised by KS with the assistance of CI, CL and SB. PubMed, PsycINFO, Web of Science and Google Scholar databases were searched between 23 April 2023 and 1 August 2023. The search strategy (Figure 2) was based on the SPIDER tool.^56^ *Sample*: Women aged 40–65 years (and their HCPs); *Phenomenon of Interest*: Barriers and enablers to SHSW services; *Design*: Peer-reviewed research (qualitative/quantitative/mixed-methods); *Evaluation*: Critical Appraisal Checklists (CASP) criteria^57^; *Research type*: Systematic review to optimise identification of relevant articles. The detailed search strategy is documented in Figure 2. Search limits included: English language and adults and middle-aged adults. No date or country of setting limits were applied. The included articles’ reference lists were hand-searched for additional relevant articles. Articles identified in the search were exported to EndNote. After removal of duplicates,^45^ the remaining articles were exported to Excel.

### Selection

Titles and abstracts were screened for relevance by KS and articles that did not meet the inclusion criteria were removed. Full texts of the remaining articles were reviewed independently in Microsoft Excel by KS and CN. Any disagreements were resolved through discussion between KS, CN and CI to reach a final decision.

### Data extraction

Study characteristics were extracted by KS using a pre-set proforma on Excel (author, title, year published, participant characteristics, study aim.) Further study characteristics were then extracted by hand by KS onto a table in Microsoft Word (participant characteristics: sample, age range, gender of participants, relationship status, sexual orientation, language requirements, education level, ethnicity, study design and study findings).

### Quality assessment

The CASPs^57^ were used to systematically assess the trustworthiness, relevance and results of the articles. The appropriate checklist was used for each study, for example the qualitative checklist was used for qualitative studies. The reporting of the results of the study were assessed for validity, what the results were, and whether the methodology was sound. KS rated the studies (high, moderate or low quality) using the checklists as guides. About 10% were randomly selected and rated by a second author (CI, SB, CL, CN) to ensure validity. Any discrepancy in the rating was discussed, and if required, a third or fourth author would have been consulted to ensure unanimity. However, there were no discrepancies, resulting in a Cohen’s Kappa rating^51^ of 1. Eligibility of articles was not determined by quality rating.

### Data synthesis

The socio-ecological model (SEM)^52^ was used to conceptualise and organise the findings into the enablers and barriers to accessing SHSW care, in terms of intra-personal factors, interactions with providers, organisational factors, community factors and public policy. The use of the SEM model has precedence in contextualising the complex levels of socioecology in which individuals are embedded, and the social, structural and cultural influences on behaviours affecting access to SHSW care. In the past, it has proved valuable in the analysis of the barriers and enablers to SHSW care for female sex workers,^58^ adolescents,^59^ urban men^49^ and migrant Asian women.^50^ Narrative synthesis was used to explore relationships within and between study findings. This method was employed because characteristics of the study designs and outcomes were too heterogenous to yield a meaningful summary of findings using a meta-analysis. The main findings and conclusions were grouped and coded inductively into descriptive themes that emerged from the data within the categories of ‘barriers to’ and ‘enablers of’ access to SHSW care, as defined by the inclusion criteria (Table 1). Data were further grouped and coded to iteratively develop and refine descriptive themes, with each study contributing new themes, using the SEM. Generation of higher-level analytical themes was undertaken.

## Results

### Study characteristics and main findings

Eighty-one studies were included in the final selection (Table 2).

**Table 2.** Quality assessment of studies for the barriers and enablers to accessing sexual health and sexual well-being services for midlife women (aged 40–65 years) in high-income countries: A mixed-methods systematic review.

Author (year of publication), study location	Study population characteristics (sample, age range, gender of participants, relationship status, sexual orientation, education level, ethnicity)	Study design and methodology	Aim of study	CASP quality	What are the results?
Auerbach et al. (2020), USA^54^	43, 55.6% were aged 41–55 years, 25 women who identified as cisgender, 10 women who identified as transgender, 3 women who identified as ‘other’, 55.5% more than high-school education, 71% Black/African/Afro-Caribbean, 5 HCPs (one identified as a transwoman, two as cisgender women, two as cisgender men)	Qualitative. Focus groups. Convenience sampling.	Barriers and facilitators to including transwomen in HIV treatment and support services traditionally focused on cisgender women.	High, good mix of participants.	Histories of trauma, need for space safe away from men, unique challenge of being a woman in the world. Lack of understanding that many cisgender women are receptive to education, with a low barrier to improving their knowledge. Drawn to women’s care spaces out of desire for community, care tailored to specifically meet women’s needs, and a space that feels free of stigma and harm. Inclusive all-women care environment would disentangle transwomen demographically from men who have sex with men, foster community and understanding, affirm gender.
Bass et al. (2022), USA^60^	34, age 18–52, mean age 34–46, 21% identified as female, 82% identified as transwoman, 6% identified as queer, 2.9% identified as ‘additional’, 56% some college education and above, 44% African American, 41% White, 21% Latinx, 12% other races	Quantitative. Cross-sectional study. Convenience sampling.	Barriers and facilitators to HIV PrEP use in transgender women.	High.	Knowledge of HIV PrEP is low. Persistent beliefs about effect of PrEP on hormone use. Need for culturally appropriate trans-specific messages in HIV prevention interventions and communications.
Benyamini et al. (2008), Israel^61^	814, Aged 45–64, women, gender not specified, presumed cisgender, 540 long-term Jewish resident women, 151 Russian immigrants, 123 Arab women	Quantitative. Cohort. Questionnaire. Stratified random sampling.	Assess rates of primary and preventive healthcare use among midlife women from different cultural origins to identify socio-demographic and health characteristics that could explain differences in healthcare use.	High.	Long-term residents reported more preventive visits and screening tests compared with immigrants and Arab women. Cultural group, education, self-rated health and health motivation were significantly associated with use of primary and preventive care. Ethnic/origin group differences were mostly related to cultural rather than financial barriers. Arab women’s low use of preventive gynaecologic care related to lack of physicians of same culture and gender.
Blackstock et al. (2021), USA^62^	52, Mean age 44.7 years, 11.5% transgender women, 88.5% gender identity not specified, presumed cisgender, 34.6% less than a high school education, 86.5% Latina or non-Latina Black	Quantitative. Cohort study. Pilot intervention. Convenience sampling.	Evaluate HIV PrEP outreach and navigation intervention.	Moderate (Study aim not completely clear).	50% Aware of HIV PrEP. Gap between PrEP interest and connecting to PrEP care. Importance of peer-outreach.
Bullington et al. (2022), USA^63^	578, Aged 25–60 (35 experienced menopause aged under 41, 101 experienced menopauses aged 41–45, 442 experienced menopause aged 46–50), women, gender not specified, presumed cisgender, majority Black ethnicity but mixed ethnicity study (e.g. 9% White)	Quantitative. Cohort. Convenience sample.	Description of premature and early menopause and subsequent hormonal treatment in women living with or at risk of HIV.	Moderate (significant confounding factors as to why older women received less hormonal therapy).	51% of women who experienced menopause before 41, 24% of women 41–45, 7% of women between 46 and 50 received menopausal hormonal therapy or hormonal contraception. Women Living with HIV are less likely to access hormone therapy for menopause than other women.
Bush et al. (2007), USA^64^	22, Physicians	Qualitative. In-depth interviews. Convenience sampling.	Physician’s perceptions on HRT and counselling strategies.	High.	Many felt that women should oversee HRT decision. Decision aids are needed to guide discussions with women.
Cahill et al. (2020), USA^65^	19, Mean age 42, 72% had high-school or less education, identify as transgender women, mixed ethnicity study	Qualitative. Focus groups. Convenience sampling.	Explore barriers to HIV PrEP uptake.	High.	Some had not heard of HIV PrEP, some reported their HCPs had not told them about PrEP, some had questions about side-effects, some expressed medical distrust. Expressed need for gender-affirming healthcare services, concerns about interactions between feminising hormones and PrEP, these interactions need to be made clear in health education campaigns. Clinicians should be trained in providing gender-affirming care.
Cannon et al. (2018), USA^66^	68, Ages 18–50 but ages 35–50 analysed separately, women, gender identity not specified, presumed cisgender, 59% African-American, 25% White, 14.9% other	Quantitative. Cross-sectional study: survey with multiple-choice close-ended questions administered via interview. Convenience sampling.	Risk of unintended pregnancy among newly incarcerated women.	Moderate (small study).	Poor knowledge about emergency contraception safety, when and how to take. About 58.8% aged 35–50 used no contraception at intake, but 63.2% would accept free contraception at release.
Cappiello and Boardman (2021), USA^67^	35, Nurse practitioners in their first 2 years of community-based practice	Qualitative. Interviews. Convenience sampling.	Exploration of perceived competency in providing sexual and reproductive healthcare.	High but small study.	More preparation needed to discuss STIs, dyspareunia and pelvic pain. Interest in discussion of sexuality across the lifespan and postmenopausal sexual concerns.
Clarke (2009), USA^68^	6 Magazines, 3 for middle-aged women, all 3 have circulation of >2,000,000	Qualitative. Exploratory content analysis.	Portrayal of sexuality, sexual health and sexual disease.	High.	Major themes: sex depicted as a woman’s work and responsibility in a marriage; accountable for husband’s sexual fulfilment, and happiness. Fear-mongering in refusing husband’s request for sex. Idea that sex can be viewed as a workout and beneficial for women’s health.
Cronin et al. (2023), England, Finland, Denmark, New Zealand, Australia and USA^22^	48 Nurses, ages 37–65 (majority over 40 years)	Qualitative. Focus groups and interviews.	Perspectives of nurses (wide range of clinical settings) on digital interventions as strategies to support menopausal women. To understand the requirements for designing health interventions for support in the workplace.	High.	Women were frustrated by the stigma and lack of recognition and awareness at work about menopause. Without awareness, women felt uncertain of the validity of their own experiences, often questioning themselves and their concerns. Colleagues were judgemental, lacked insight, tiredness related to menopause symptoms was not socially acceptable. Participants from many countries felt that many women were unprepared and there was a lack of knowledge about the menopause. Mothers had not prepared them – no detail/depth in explanations. ‘You just get on with it, that sort of thing’. ‘I talk to mums everyday about everything to do with their bodies and their bleeding, babies and everything . . . there are so many variations on how it is for premenopausal women, like maybe we don’t really understand enough’. Participants preferred to select from a range of interventions available across different formats (to meet individual needs). Participants had spent a long time looking for information, with poor results. They felt that due to the widespread use of smart phones, it should be possible to have quick, easy access at any time and anonymously, to a private, secure platform with evidence-based information. They felt that digital resources should be culturally sensitive, with podcasts, videos, virtual reality, mindfulness, expert blogs.
Dakshina et al. (2014), UK^69^	202, Ages 16–60, Median age 41, IQR 35–49 years, women, gender identity not specified, presumed cisgender	Quantitative. Cross-sectional study. Purposive sampling.	Ascertain proportion of women with a documented discussion about their family planning needs within a 12-month period.	High.	Contraception only documented to have been offered to 15% of women who required it.
Dalrymple et al. (2017), Scotland^70^	31 (18 Women, 13 men), ages 45–65, women, gender not specified, presumed cisgender, most had been divorced, most from deprived areas as per Scottish Index of Multiple Deprivation areas	Qualitative. Interviews. Convenience sample.	Psychosocial factors that influence risk-taking.	High (but small study and may not have been representative due to recruitment from clinics and sporting centres and limited ethnic diversity).	Reassured if new partner had few previous partners, long previous relationships, or had recently spent time alone. Very few used condoms or tested for STIs. Unwanted pregnancy shaped risk perceptions through life-course. Prioritisation of intimacy above risk of STIs. Feelings of reassurance and safety from STIs were associated with intimacy. Motivation to seek intimate and monogamous relationships, and a sense of emotional connection with new partner, lent safety to actual exposure to STIs. Vulnerability associated with relationship transitions – guilt and loss of self-esteem leading to prioritisation of emotional rather than health needs (safe sex). Social norms relating to age-appropriate sexual and health seeking behaviours – older people ‘should know better’, ashamed to seek help. Distancing from young people’s risky behaviour. Felt had to adjust to new sexual culture – fast progression to sex in new relationships. Sense of freedom linked with feeling young, encouraging more risk taking.
Dietz et al. (2018), USA^71^	30, Aged 45–60, women, gender identity not specified, presumed cisgender	Qualitative study. Focus groups. Convenience sample.	Women’s knowledge of menopause, preferred sources of information, barriers to treatment.	High (but not generalisable).	Feel insufficiently informed about hormone therapy. Prefer older female providers.
Doblecki-Lewis et al. (2015), USA^72^	8, Aged 40–59 (analysed separately from other women in study aged 18 to >60), women, gender identity/education/ethnicity not separated out for this age group, but study included predominantly Black and Hispanic participants and 77% of total participants were heterosexual)	Quantitative. Cross-sectional study. Purposive sampling.	Self-reported sexual risk behaviour, awareness and perception of HIV Pre exposure Prophylaxis (PrEP) and HIV Post exposure Prophylaxis (PEP) in a sheltered population (women experiencing homelessness).	High (but although analysed separately, small sample of midlife women).	Women who reported that they would ‘definitely consider’ or ‘might consider’ PrEP or PEP were significantly younger (mean age = 29.3 years) compared with those who reported they would ‘definitely not consider’ these strategies (mean age = 41.5 years; *p* = 0.004 for difference).
Donati et al. (2009), Italy^73^	720, Aged 45–60 years, women, gender identity not specified, presumed cisgender	Quantitative. Cross-sectional study. Purposive sampling.	Women’s attitude, knowledge and practice regarding menopause and HRT.	High.	Minority received information about menopause, those who did found it poor and contrasting.
Duff et al. (2018), Canada^74^	109, Aged 44–53, women, gender identity not specified, presumed cisgender, 33% Lesbian, gay, bisexual, queer, two-spirit, 90% high school graduate, 56% Indigenous, 7% African/Black/Caribbean, 36% White	Quantitative. Cohort study. Convenience sampling.	Correlates of antiretroviral adherence among perimenopausal and menopausal women living with HIV.	High.	Severe menopausal symptoms, injecting drug use and physical/sexual gender-based violence associated with <95% antiretroviral adherence.
Du Mont et al. (2021), Canada^75^	27, HCPs who look after people who have been sexually assaulted	Quantitative. Cross-sectional. Convenience recruitment.	Evaluation of networks’ capacity to address needs of trans-sexual assault survivors.	High.	Barriers to providing care: lack of knowledge, discriminatory attitudes, limited training opportunities. About 100% felt would benefit from further training to help trans-people. Hospital policies not trans-sensitive. Paucity of trans-positive services for referral/collaboration. Facilitators: efforts to promote trans-positive environments, trans-positive hospital policies, partnership building with other services.
Ejegi-Memeh et al. (2021), UK^76^	10 (4 Aged 40–65, results separated out), aged 50–83, women, gender identity not specified, presumed cisgender	Qualitative. Interviews. Convenience sampling.	Barriers and facilitators to sexual discussions in primary care in women with type 2 diabetes mellitus.	High quality but small number.	Problematic changes to sexual health and well-being, attributed to diabetes, menopause, ageing and changes in intimate relationship, tended not to be discussed with HCPs. Women assumed HCPs did not broach topic of sex due to embarrassment, ageism, social taboos around older women and sex. Facilitators: professional-patient rapport, female HCPs, instigation of conversation by HCP.
Erens et al. (2019), UK^8^	23 (5 Women aged 40–65 years, results separated out), aged 55–74, women, gender identity not specified, presumed cisgender, 3 married, 1 widowed, 1 co-habiting, 30,503,880 (9,751,175 women aged 40–65 years, results separated out)	Mixed methods. Qualitative. Interviews. Purposive sampling from nationally representative sample (Natsal-3). Quantitative. Cross-sectional (nationally representative sample from private residential addresses, Natsal-3).	To explore how older people see their health status as having influenced their sexual activity and satisfaction; and to further understanding of how they respond and deal with the consequences.	High.	Where help had been received, it was in the form of sildenafil for men. No participants in the qualitative component mentioned any other form of help. Women described continuing to have sex despite a health-related reduction in their own sexual desire to ensure the continued stability of the relationship. ‘You get used to what you like and because I can’t have what I like I’m not really bothered’. After adjusting for age and relationship status, the odds for being sexually active were much lower for those who saw themselves as being in bad/very bad health compared with those in very good health. For women in a steady or cohabiting relationship, after adjusting for age, satisfaction with their sex life was associated with feeling happy in the relationship.
Erickson et al. (2019), Canada^77^	292, mean age 43, 10% transgender women, 90% cisgender women, 48% had completed high-school, 58% indigenous, 34% White, 4% Afro-Caribbean, 5% other	Quantitative. Cross-sectional. Convenience sample.	How incarceration affects HIV treatment outcomes.	High.	Exposure to recent incarceration independently associated with reduced odds of HIV virological suppression.
Esposito (2005), USA^78^	56 (40 Women, 6 HCPs), ages 39–62, women, gender identity not specified, presumed cisgender, 38% married or cohabiting, 6, HCPs, ages 34–56, all White and European American	Qualitative. Focus groups. Convenience and snowball sampling.	Immigrant Hispanic and Healthcare Providers expectations of healthcare encounters in the Menopause.	High.	Linguistic ability. Cultural inhibitions to not bringing up topic. Dealing with American providers required higher level of assertiveness than previously needed. Lack of access to advice – some knowledgeable relatives were now dead, others never talked about menopause/could not remember it/denied symptoms. Brochures and videos nonspecific – unsure if applied to them, failed to address intensity of symptoms. Women wanted provider-initiated, individualised, anticipatory guidance for menopause. Providers felt other challenges (diabetes, heart disease, HIV prevention) were more important. Providers did not have time to assess their needs. Need for better mainstream medical relief from menopause symptoms. HCPs questioned whether menopause an issue for immigrant Hispanic women. Symptoms were not important because women were socialised to minimise their gynaecological discomforts in context of competing family needs. May manage symptoms effectively through alternative health resources such as local medicine woman. HCPs wanted patients to be informed and clear about what they wanted. Frustrated with lack of continuity in care, for example, when international mobility complicated care. Cultural expectations of stoicism around gendered issues such as menopause.
Ettinger et al. (2000) and Ettinger and Pressman (1999), USA^79,80^	749, Aged 50–65, women, gender identity not specified, presumed cisgender	Quantitative. Cross-sectional. Convenience sampling.	Predictors of women receiving HRT counselling.	High.	Higher level of education and higher level of income associated with increased likelihood of HRT counselling being obtained.
Fox-Young et al. (1995), Australia^81^	40, Aged 45–55, women, gender identity not specified, presumed cisgender	Qualitative. Focus groups. Convenience sampling.	Women’s perceptions and experiences of menopause, HRT, doctor–patient relationships.	High (but not generalisable).	Lack of reliable and accurate information about menopause. Need to foster open discussion between women and HCPs, need for equal partnership.
Gazibara et al. (2022), Spain and Serbia^82^	461 from Madrid, 513 from Belgrade, 40–65, Women, gender not specified, presumed cisgender	Quantitative. Cross-sectional study. Convenience sampling.	Compare climacteric symptoms associated with health-related quality of life.	High (unclear whether similar level of contra-indications in two different populations).	Similar ratings of somatic and urogenital complaints. Serbian women of higher education were more likely to use HRT.
Gkrozou et al. (2019), UK^40^	22 Apps	Review analysis of menopause apps.	Identify mHealth apps that address the menopause with a focused view on the degree of medical professional involvement and evidence base practice in their design.	High (but limited due to small number).	Only 22.7% of the apps analysed had documented evidence-based practice in the form of guidelines or treatment protocols.
Gleser (2015), UK^83^	62, Specialist trainee doctors	Quantitative. Cross-sectional study. Questionnaire. Convenience sampling.	Determine views and attitudes of specialist trainee doctors towards sexual health in the (peri)menopause.	Moderate. (Did not look at confounders such as ethnicity, sexuality).	Do not believe that women proactively raise their concerns regarding menopausal symptoms affecting sexual health without being asked. Reported lack of consultation time as main barrier to address sexual health and sexual problems in menopausal women. Some reported perceived unease from patient’s side, poor coverage of topic in curriculum, lack of training.
Gomez-Acebo et al. (2020), Spain^84^	2038, Ages 20–85 (1031 women born before 1950 had average age of 70, 1007 women born after 1950 had average age of 48), women, gender not specified, presumed cisgender, majority with low level of education, 12 Spanish provinces	Quantitative. Cross-sectional design. Purposive sampling.	Influence of individual and contextual socio-economic levels on reproductive factors.	Moderate (did not stratify by ethnicity).	For women born before 1950: more educated women double use of HRT, women living in less vulnerable areas double use of HRT. But socio-economic inequalities in HRT not true for women born after 1950.
Goparaju et al. (2015), USA^85^	39, Median age 49, women, gender identity not specified, presumed cisgender, Majority Black/African American or Latina/Hispanic, mixed level of education, 50% married	Qualitative. Focus groups. Purposive sampling.	Women’s knowledge, attitudes and potential behaviours regarding HIV PrEP.	High.	PrEP awareness was extremely low and the HIV-negative women urged publicity: they wanted to use and recommend it to others despite potential side effects, and difficulties with access, duration and frequency of use. Women’s reactions to PrEP differed based on their sero-status: HIV-negative women expressed much enthusiasm while the HIV-positive women voiced caution and concerns based on their experience with ARVs.
Guo and Sims (2021), USA^86^	498, Aged 42–65, women, gender identity not specified, presumed cisgender, Chinese American women	Quantitative. Cohort study. Purposive recruitment.	Patient-level factors associated with Pap uptake.	High.	Having a female healthcare provider positively associated with Pap test uptake. Not having a primary healthcare provider and not having time to go to the doctor negatively associated with Pap test uptake.
Heinemann et al. (2008), USA^87^	10,297, Age: 40–70, women, gender not specified, presumed cisgender, education level: over 60% were low/medium, majority married/stable relationships, 9 countries on 4 continents	Quantitative. Cross-sectional. Validated survey. National representative population panes and quota sampling.	Prevalence of menopausal women’s symptoms, motivations for hormone therapy.	Moderate (ethnicity not described).	Self-reported symptoms did not differ significantly among women in Europe, North America, Latin America and Indonesia. Prevalence range of hormone therapy from 50% to 1.8%. Acceptance of HRT affected by: worry about side effects.
Hendren et al. (2019), Canada^88^	154, Median age 41–45, 53% women (Adult nephrologists)	Quantitative. Cohort. Convenience sampling.	Adult nephrologists training in, exposure to and confidence in managing women’s health.	High.	65% lacked confidence in women’s health issues, 89% felt that interdisciplinary clinics and continuing education seminars would improve their knowledge.
Hillman et al. (2020), UK^89^	142,919,989, Aged over 40 (2,677,613 HRT prescriptions), women, gender identity not specified, presumed cisgender, from 6478 practices	Quantitative. Cross-sectional study. Purposive sampling.	General practice HRT prescription trends and their association with markers of socio-economic deprivation.	High.	HRT prescribing rate 39% lower in practices from most deprived quintile compared to most affluent. After adjusting for cardiovascular risks/outcomes, 18% lower. More deprived practices have significantly higher tendency to prescribe oral HRT (should be avoided if high cardiovascular risk).
Hinchliff et al. (2021), UK^90^	23 (12 Women, 11 men), ages 58–75, women, gender identity not specified, presumed cisgender, majority married	Qualitative. Interviews. Purposive sampling.	Explore why older adults do, or do not, seek help for sexual difficulties.	High.	Both women and men framed help-seeking in relation to erectile dysfunction. Reluctance/inability to talk about sex with partner stopped them getting help. Feeling embarrassed to talk about sexual difficulties – considered taboo. Dealing with other health conditions more important. Significant barrier was concern about interaction of medicines prescribed for the sexual difficulty with those already taken for health conditions. Spent a long time, sometimes years, thinking about whether to get help. Patient fear of not being taken seriously and doctor reticence to ask. Help-seeking journeys often ended without resolution. Most were not asked about sexual well-being even though had conditions known to affect sex life. Doctors prioritised general health over sexual health. Having access to approachable doctor facilitated help-seeking. Patients saw help-seeking only appropriate up to a certain age.
Howells et al. (2019), UK^91^	73, Women, gender identity not specified, presumed cisgender, 56% Black African, 5% Black Caribbean, 5% Black British, 3% White British, 10% White other	Quantitative. Cross-sectional study. Retrospective case note review. Purposive sampling.	Assess uptake and effectiveness of HRT in postmenopausal women living with HIV.	High.	Good symptom control (91%) in those of Black ethnicity who started HRT but low uptake (52%).
Huang et al. (2009), USA^92^	1977, Aged 45–80 (77% between 45 and 64), women, gender identity not specified, presumed cisgender, 67% currently married/living as married, 44% White, 29% African American, 18% Latina, 19% Asian	Quantitative. Cohort study. Purposive sampling.	Factors influencing sexual activity and functioning.	High.	43% Reported moderate to very high sexual desire or interest, decreased with increasing age varied according to race/ethnicity/co-habitation. Sexual desire and interest strongly associated with sexual satisfaction. About 40% reported problems with sexual activity. Physical and mental health strongly associated with sexual desire and interest. Lack of partner capable of or interested in sex important.
Huynh et al. (2022), USA^93^	87, Ages <45 (40%), 45–65 (44.8%), >65 (12.6%), women, gender identity not specified, presumed cisgender, 85% Heterosexual, 72% married or partnered, 82% Caucasian, 5.7% Asian, 4.6% Black or African American, 1% Latina, 1% American Indian or Alaskan, 4.6%, Other/prefer not to say, Focus groups: 13, Individual interviews: 3	Mixed methods. Cross-sectional study and Qualitative (interviews and focus groups). Convenience sampling.	Characterise education that patients with breast cancer receive about potential sexual health effects of treatment, and preferences in format, content, timing.	Moderate quality (? Selection bias and homogenous population, and qualitative element very small).	Few women received information about sexual health effects of treatment. Patients in favour of multiple options of education being offered, with emphasis on in-person options and support groups.
Jacobs et al. (2014), USA^94^	3258, aged 42–52, women, gender not specified, presumed cisgender, 7 US sites, 68% married, 76% had some form of college education, 28% African American, 7% Chinese, 8% Japanese, 7% Hispanic, 50% Caucasian	Quantitative. Cohort. Mixed recruitment methods.	Relationship between perceived everyday multi-ethnic/multiracial and other discrimination and receipt of cervical (and breast) screening.	High (but difficulty with concept of ‘reported discrimination’).	Reported discrimination owing to physical appearance and gender associated with reduced receipt of Pap smear regardless of race. Perceived discrimination much higher in African American women.
Kerkhoff et al. (2006), Ireland^95^	58, Age 22–81, median age 46, women, gender identity not specified, presumed cisgender	Quantitative. Cross-sectional study. Convenience sample.	Assess female renal transplant recipient’s awareness of gynaecological issues, and to assess uptake of screening services.	High (but not generalisable).	84% Aware as to why they should have regular cervical smears.
Kingsberg et al. (2013), USA^34^	3046, Age: postmenopausal, women, gender identity not specified, presumed cisgender	Quantitative. Cross-sectional study. Questionnaire. Purposive sampling.	Characterise postmenopausal women’s experiences with vulvar-vaginal atrophy and interactions with HCPs about it.	High.	24% Attributed symptoms to menopause, 65% had discussed symptoms with HCP, 40% using treatment (62% who had consulted HCP were using treatment). Concerns about side effects and cancer risks limited access to treatment.
Levin et al. (2023), Israel^96^	252, Mean age 54, women, gender not specified, presumed cisgender	Quantitative. Cross-sectional study. Convenience sample.	Compare stage and survival of cervical cancer of Jewish and Arab women.	Moderate- (many more Jewish women in study, unsure re. power, and many confounders).	Arab women at higher risk to be diagnosed with advanced cervical cancer than Jewish women.
Lewis et al. (2020), UK^24^	19 (10 Women, 9 men), ages 40–59, women, gender not specified, presumed cisgender	Qualitative. Face-to-face interviews. Purposive selection from NATSAL-3.	Identify factors shaping STI risk perceptions and practices among individuals contemplating or having sex with new partners following end of long-term relationship.	High (but small number).	Loss of fertility reduces motivation to prioritise safe sex. Inadequate indicators to assess STI status of partners, for example, demeanour, appearance. Self-perception as low risk even if have come out of long-term relationship. Constraints on the navigation of sexual safety include self-perceived STI risk rooted in past. Perceived dominance of ‘couple culture’ pressure to re-partner. Perceived norms vary between social networks – normalisation of condomless sex with new partners, paying for sex, ‘othering’ of those at risk of STIs/HIV. Intersecting gender-age dynamics in negotiation of risk prevention strategies – association with youth, lack of familiarity, concerns about erectile dysfunction. Enablers: peers and younger relatives’ influences. Age-related barriers to accessing condoms; disconnection from safe sex messaging and services (men who have sex with men and youth focused). Greater public discussion about sexuality and more positive representation of sexuality in mid-life in tension with sensationalised media coverage. Age-gender barriers to accessing condoms in shops/pharmacies.
Lippman et al. (2016), USA^97^	50 (11 Gave in-depth interviews), median age 42, transgender women 56% post high-school education, 30% Black African-American, 22% White/Caucasian, 20% Hispanic/Latino, 8% Asian/Pacific Islander, 8% Native American, 12% multi-racial	Mixed. Quantitative. Cross-sectional. Qualitative interviews. Convenience sampling.	Feasibility and acceptability of HIV self-testing.	High.	68% Prefer HIV self-testing to attendance at clinic. Emotional support embedded into service very important.
Maar et al. (2013), Canada^98^	18, HCPs	Qualitative. Interviews. Convenience sampling.	Structural barriers to cervical screening.	High.	Shortage of HCPs, lack of recall system, geographic/transportation barriers, health literacy, socio-economic inequalities, generational effects, colonial legacy.
Maiorana et al. (2021), USA^99^	67, 36 Staff, 31 participants in interventions, average age 44 (range 21–63), all participants identified as transgender women of colour, 77% of staff identified as transgender women of colour	Qualitative. Interviews. Convenience sampling.	Identify commonalities amongst intervention services for Transgender Women of Colour (addressing unmet basic needs, facilitating engagement in HIV care, health system navigation, improving health literacy and support) and relationships formed during implementation.	High.	Interplay of transphobia, sexism and racism. Transgender women have low self-esteem, feel defensive and socially excluded due to stigma, and previous experiences of marginalisation in community and healthcare system. Gender-affirming services and caring relationships help to engage transwomen of colour in care. Intervention services need to be complex, and HCPs need to be able to perform many roles. Services that address basic unmet needs such as food, housing before addressing other barriers to engaging in HIV care.
Moro et al. (2010), Canada^100^	4, Age 46–72, women, gender-identity not specified, presumed cisgender	Qualitative. Focus group. Convenience sampling.	Enablers and barriers to obtaining bioidentical hormones and how to improve the access path.	Poor (small number).	Seek information from friends/books/websites/HCPs. Quality of available information a barrier to making informed decision. Internet a confusing medium-wide range of quality. HCP with supportive/trustworthy/ample information seen as key enabler to care. Some physicians did not listen to their needs. Some made unilateral decisions favouring HRT without consulting patient. Struggle to access physicians who would prescribe HRT. Cost of bioidentical HRT big barrier. Suggestions: continuing education for physicians, comprehensive website with risks and benefits of HRT, regular seminars for menopausal women, policy change to allow other health professionals to prescribe HRT, women-only clinics.
Munro et al. (2017), Canada^101^	14 Women (8 aged over 40) and 10 HCPs, women who identify as transgender women	Qualitative. Interviews. Convenience sampling.	Dynamics of healthcare utilisation by transwomen living with HIV.	High, but small study.	Importance of coordinating HIV services and transition-related care. Importance of training service providers.
Nappi and Kokot-Kierepa (2010), USA^102^	4246, Aged 55–65, women, gender identity not specified, presumed cisgender, Sweden, Finland, UK, USA, Canada	Quantitative. Cross-sectional. Interviews using structured questionnaire. Purposive sampling.	Issues related to vaginal atrophy via an international survey.	Moderate (purposive sampling but not sampled to be representative).	Different needs in different countries (51% in US versus 10% in Finland aware of local treatment); country-specific approaches to improve uptake of treatment for vaginal atrophy. About 77% believe women uncomfortable discussing vaginal atrophy, 42% not aware that local treatment is available.
Nemoto et al. (2021), USA^103^	60 in Cohort study (34 enrolled in HIV care and 26 who had never enrolled), 12 interviews, age range 19–60, average age 41, transgender women (78% gender identity transgender woman, 21% female), 32% college or above level education, 80% African American, 13% Hispanic, 2% Asian, 5% Multiracial	Mixed study: Cohort and qualitative. Interviews. Convenience sample.	Describe access to HIV primary care for African American transwomen living with HIV.	High.	Transphobia and community stigma are strong barriers to enrolling in HIV care; 44% who had enrolled in HIV care had not received anti-retroviral treatment (ART); 50% of those on ART had poor adherence. If engaged in sex work, sold drugs, or experienced higher levels of transphobia less likely to have enrolled in HIV care.
Nusbaum et al. (2004), USA^104^	1196, 54% aged <45, 15% 45–54 years, 32% aged over 54, women, gender identity not specified, presumed cisgender	Quantitative. Cross-sectional study. Survey. Convenience sampling.	Self-reported sexual concerns and interest and experience in discussing these concerns with physicians.	High.	96% of ‘middle aged’ women showed interest in discussing with HCP. Women who discussed concerns found it helpful. Felt that discussing would be easier if physician broached topic. Having female physician would facilitate discussion. Most women had never had topic raised by physicians.
Okhai et al. (2020), UK^105^	6455 Women (1595 aged 40–50, analysed separately, 607 aged >50, analysed separately), women, gender identity not specified, presumed cisgender, all acquired HIV via sex with men, 14% White, 5.7% Black Caribbean, 70.6% Black African, 3.6% Black other, 2.7% South Asian, 3.4% Mixed	Quantitative. Cross-sectional study. Purposive sampling.	Investigate whether association between menopausal age and engagement in HIV care.	High, large study.	Postmenopausal aged women less likely to be engaged in HIV care than younger ages differs from previous studies? Due to different definitions of engagement in care. However, suggestion that better adherence to antiretrovirals among 40–50 years than younger women.
Ong et al. (2022), Australia^106^	3 Suburban GP practices, qualitative: 33, 17 doctors, 7 nurses, 9 administrative staff	Mixed methods. Quantitative. Cross-sectional. Qualitative. Interviews. Convenience sample.	Hub and spoke model: primary care HIV/STI testing and treatment with support of specialist SH centre hub.	High.	Early and sustained increase in chlamydia, gonorrhoea, syphilis, HIV testing. Many training needs identified by GPs, nurses and admin staff, for example, taking a non-judgemental sexual history, using culturally appropriate terms, approaching partner notification, legal requirements, how to invite patients to talk, work flow in the clinic, to provide flexible time for lectures, routine refresher training-enthusiasm to upskill, rarely provided consultations with ‘priority’ populations (LGBTQ, sex workers, people who inject drugs, certain ethnicities, incarcerated, refugees).
Parkes et al. (2020), UK^107^	7019, Ages 16–74 but approximately 50% over 40, and ages analysed in groups, women, gender identity not specified, presumed cisgender, 88% White	Quantitative. Latent class analysis from NATSAL-3. Purposive sampling.	Identify clusters of sexual health markers with their socio-demographic, health and lifestyle correlates.	High.	6 Classes found for women, for example, 7% ‘unwary STI risk takers’.
Politi et al. (2009), USA^108^	40, Mean age 55, women, gender identity not specified, presumed cisgender, unmarried women, 73% college degree, 98% White	Qualitative. Interviews. Convenience sampling.	Describe the experience of middle aged and older aged unmarried women’s communication with HCPs about Sexual Health.	Moderate (not generalisable).	Sexual minority women hesitant to share information due to previous negative experiences when disclosing sexuality. Unmarried women more likely to disclose information about sexual health if perceive HCP does not make assumptions and appears non-judgemental. Unmarried women more comfortable talking to female providers about sexual health.
Reese et al. (2020), USA^109^	144, Mean age 56, women, gender identity not specified, presumed cisgender, 62% partnered, 67% White, 27% Black/African American, 4% Hispanic/Latina	Quantitative. Cross-sectional study. Web-based baseline self-report surveys. Convenience sample.	How commonly women sought help for sexual health concerns after breast cancer treatment and from whom, and whether help-seeking was associated with sexual function/activity/self-efficacy.	High.	Women seeking help were younger and more likely to be partnered; 42% sought help from intimate partners, family/friends; 24% from HCPs, 19% from online/print materials. Women seeking help from outlets other than HCPs had significantly lower self-efficacy than those who did not.
Roberts and Sibbald (2000), UK^110^	181 General practitioners, 147 Practice nurses	Quantitative. Cross-sectional study. Purposive sampling.	Knowledge and views about HRT.	Moderate	57% of GPs and 85% of practice nurses wanted more knowledge about HRT.
Rostom et al. (2002), Canada^111^	51, Ages 40–70, women, gender-identity not specified, presumed cisgender, 75% had college or University degree	Quantitative. Randomised trial.	Perceptions of HRT associated risks, barriers to HRT, knowledge of menopause symptoms, decision aids for HRT/menopause.	High.	Realistic expectations score of risk perceptions (heart disease, hip fractures, breast cancer): 35%. Baseline knowledge score: 77%.
Samuel et al. (2014), UK^112^	124, Age range 50–75 years, mean age 52, women, gender identity not specified, presumed cisgender, 83% Black ethnicity	Quantitative. Cross-sectional study. Notes review. Purposive sampling.	Review of care provided to HIV-positive women aged over 50.	High.	71% Late diagnoses, 42% with advanced/very advanced HIV, 22.6% documented missed opportunities for early diagnosis.
Sangaramoorthy et al. (2017), USA^113^	35, Age: 40–71, 88% between 40 and 59 years, women, gender not specified, presumed cisgender, 32% college or higher education, 28 native-born African Americans, 7 Black African participants	Qualitative. Interviews. Purposive and snowball recruitment.	Perceptions and experiences of HIV-related stigma.	High.	Intersectional stigma is a central feature in lives, at interpersonal, familial/community and institutional/structural levels. Negative responses to gender, age, race and disease.
Schneider (1997), France, Germany, Spain, UK^114^	929, Aged 40–65, women, gender identity not specified, presumed cisgender	Quantitative. Cross-sectional. Convenience sampling.	Attitudes towards and use of HRT.	High.	Use of HRT: 18% Spain, 55% France; 38%–61% of women had not discussed menopause or symptoms with HCP; 66% women believed they needed more information about HRT.
Sevelius et al. (2014), USA^115^	58, 20 interviewees, 38 focus group participants, 85% aged 40–59, women who identified as transgender women, 85% African-American/Black, 5% Pacific Islander, 5% Multiracial, 5% Caucasian	Qualitative. Interviews and focus groups. Convenience sample.	Barriers and facilitators to engagement in HIV care among transgender women.	High (triangulation across methods).	Mental health problems, substance use, poverty, transphobia, barriers to care. Avoidance of healthcare due to stigma and past negative experiences, prioritisation of hormone therapy, concern about interactions between hormone therapy and anti-retroviral. Culturally competent, transgender sensitive healthcare powerful facilitator.
Sillence et al. (2023), UK^116^	18 Menopause apps	Qualitative and quantitative. Review analysis.	Quality, feature and written review analysis of menopause apps.	High.	Data reports and visualisations empowered app users to seek out help and facilitated conversations with HCPs. Apps with clear links to HCP support, were viewed positively by app reviewers and all three apps with HCP support had ‘good’ quality scores. Few of the menopause apps were explicitly supported by HCPs. Many apps scored poorly in relation to the credibility of the source.
Simon et al. (2013), USA^117^	3530, Age 55–65 years, women, gender identity not specified, presumed cisgender	Quantitative. Cross-sectional study. Convenience sample.	Postmenopausal women’s knowledge of, and attitudes towards, vaginal atrophy.	High.	63% Associated vaginal symptoms with menopause; 80% considered vaginal atrophy to negatively affect their lives; 40% Waited 1 year before consulting HCP.
Sinko and Saint Arnault (2020), USA^118^	21, Aged 20–81 (9 aged over 41–50+ and 4 aged 31–40), women, gender identity not specified, presumed cisgender, 78% college degree or higher, 86% Caucasian, 11% African American, 1% Asian	Qualitative. Survivor narratives. Convenience sampling.	Explore nature of Gender-based violence healing.	High.	Contextual factors that influence healing: societal expectations, social reactions to self-disclosure, normalisation of violence. Internal themes of shame, self-doubt, self-blame, fear of judgement. Healing: reconnecting with self (reclaiming identity, managing symptoms, regaining control); reconnecting with others (sense of belonging, relating to others); reconnecting with world (releasing negativity, living a purposeful life).
Solomon et al. (2021), UK^119^	661, Aged 45–60, median age 49, women, gender identity not specified, presumed cisgender, 44% completed University, 72% Black African, 8.4% White UK, 19.4% other	Quantitative. Cross-sectional study. Convenience sampling.	Association between HIV clinic attendance and ART adherence and menopausal symptoms.	High, large study, majority Black African.	Severe menopausal symptoms associated with suboptimal ART adherence and HIV clinic attendance.
Strohl et al. (2015), USA^120^	215, Age range 18–70, average age 50, women, gender not specified, presumed cisgender, 22% married, 38% college graduate or above education, 100% African American/Black	Quantitative. Cross-sectional study: survey. Convenience sampling.	HPV knowledge and awareness.	High (but not generalisable (100% African American/Black)).	Knowledge of HPV, cervical cancer and HPV vaccination was low.
Studts et al. (2012), USA^121^	345, Aged 40–64, women, gender identity not specified, presumed cisgender, 61.2% married, 95.1% White	Quantitative. Randomised controlled trial, single-blind, two-arm.	Effectiveness of faith-based lay health advisor intervention to improve cervical screening rates in an area of low screening rates.	High (but homogenous study population).	Significantly more likely to have smear if baseline report of recent prior smear.
Studts et al. (2013), USA^122^	345, Ages 40–64, mean age 51, women, gender not specified, presumed cisgender, 64% High-school education or less, 61% Married, 95% Non-Hispanic Caucasian	Quantitative. Cross-sectional study: 88-Item questionnaire (built using qualitative research). Recruited from Churches. Convenience sampling.	Barriers to cervical cancer screening.	Moderate (results not generalisable as recruited from churches and homogeneous ethnicity).	Fear, worry and embarrassment prevented screening. Erroneous beliefs (that a person who has cervical cancer would have symptoms) prevented screening. Lowest perceived income adequacy, lowest perceived health status more likely to report fear of cancer being detected (significant emotional barrier). Access to healthcare facilities, health insurance coverage, cost of testing, limited transport options, lack of physician recommendation/interaction. More likely to screen if could use home kit.
Taylor et al. (2017), USA^123^	50, Ages 50–69, mean age 56, women, gender identity not specified, presumed cisgender, 48% more than high school education, 90% Black or African American	Qualitative. Interviews and focus groups. Convenience sampling.	Explore importance of sex and sexuality among women living with HIV to identify their sexual health and HIV prevention needs.	High.	Sexual pleasure increases with age. Sexual freedom (from the fear of pregnancy and traditional gender norms – expectations of being in a committed relationship or needing financial support, have more partners, sex when they like). Less pleasurable due to partner and relationship characteristics (partner’s inability to perform, unhappiness with relationships, trapped in sexless relationship). Changes in sexual abilities (physical limitations, increase or decrease in desire, ageing as barrier/improvement to orgasm experience, impact of comorbidities). Sexual risk behaviours (many condomless sex, relationship dynamics important for not practising safe sex). Ageist assumptions about sex lives and serostatus (belief older women should not be sexually active, younger men perceive sex with older women is lower risk behaviour-expectation they can have condomless sex) many fought against these stereotypes. STI prevention for older women living with HIV should promote ways to maintain satisfying and safe sex lives.
Thames et al. (2018), USA^124^	45, Ages 50–80 years, African-American, women, gender not specified, presumed cisgender	Qualitative. Focus groups, followed by community-based conference. Convenience sampling.	Community-based participatory research approach to analyse sexual health behaviours and mental health.	High.	Depression, loneliness and self-esteem issues reasons for engaging in high-risk sexual behaviours. Women did not feel comfortable discussing sexual practices with their physicians, partners, or friends.
Tortolero-Luna et al. (2006), USA^125^	235, 35–61 Years (62% aged between 40 and 61), women, gender not specified, presumed cisgender, 58% less than high school education, 80% married or co-habiting, Hispanic women (92% from Mexico)	Quantitative. Cross-sectional. Survey questions. Univariate and multivariate analysis. Convenience sampling.	Assess how English language use by Hispanic women affects their preferences for participating in decision-making and information seeking regarding medical care.	Moderate (difficult to distinguish between effect of language and effect of culture).	Decreased use of English language associated with less desire to participate in medical decision making. Increased use of English language may influence Hispanic women’s preferences for participating in medical decisions and their information-seeking behaviour.
Valanis et al. (2000), USA^126^	93, 311, Aged 50–79, women, gender not specified, presumed cisgender, 40 study centres with range of geographic and ethnic diversity, 85% White but attempted to recruit mixed ethnicity	Quantitative. Cohort study. Questionnaire. Convenience sample.	Compare heterosexual and non-heterosexual women on demographic characteristics, psychosocial risk factors, screening practices, health-related behaviours associated with increased risk of diseases.	Moderate (quality-lower proportion of non-heterosexuals than ideal).	In bisexual and homosexual women: lower cancer screening rates. Lesbian and bisexual women had more risk factors for reproductive cancers. Women reporting never having had sex as adults had lower Pap screening rates and lower rates of HRT use.
Van Dommelen et al. (2022), USA^127^	220, Physicians and advanced practice providers (182 OBGYN, 38 family/internal medicine)	Quantitative. Cross-sectional. Questionnaire. Convenience sampling.	Identify barriers to screening for sexual dysfunction among HCPs.	High.	Primary barrier for OBGYNs time constraints, for primary care provider not having enough knowledge.
Vazquez et al. (2007), USA^146^	440, 43% Over age of 45, 32% aged 35–44, women, gender identity not specified, presumed cisgender	Quantitative. Cross-sectional study. Survey. Convenience sample.	Women’s perceptions of issues they face with diagnosis of epilepsy.	High (but not generalisable).	31% of Women with epilepsy knowledgeable about menopause; 28% Discussed menopause with HCP but 52% wanted more info; 48% very concerned about impact of antiepileptic drugs on menopause.
Walter et al. (2004), UK^128^	40, Aged 50–55, women, gender identity not specified, presumed cisgender	Qualitative. Focus groups and interviews. Convenience sampling.	Women’s understanding of risk associated with menopause and HRT.	High (but not generalisable).	Women request unbiased, truthful, summarised, personalised, information. Barriers to optimal risk communication and decision making were lack of HCP time, GP attitudes and poor communication.
Walters et al. (2021), USA^129^	35, 31% Aged 36–50, 50% aged over 50, 32 cisgender, 3 transgender, 37% Heterosexual, 31% some college or more (education), 46% Hispanic/Latina, 46% Non-Hispanic Black, 9% Non-Hispanic White	Qualitative, interviews. Purposive recruitment.	Barriers and facilitators to HIV PrEP education intervention.	High.	51% Had previously heard about HIV PrEP (although many had behaviours that would put them at risk of HIV). Facilitators: offering other health and social services as well, women-focused approach, peer-outreach and navigation. Barriers: concerns about side-effects or interactions, concurrent health-related conditions or appointments, insecure housing and travel, caring responsibilities.
Watts and Jen (2023), USA^130^	27, Aged 39–57, women, gender identity not specified, presumed cisgender, 70% in long-term relationship, 74% heterosexual, 7% lesbian, 11% bi- or pan-sexual, 4% gender fluid, 4% asexual, 80% bachelor or postgraduate degree, 59% White ethnicity, 11% White Jewish, 15% mixed ethnicity, 1% Asian, 1% Latina, 1% no race given	Qualitative. Interviews. Purposive and snowballing recruitment.	Investigate perceptions and interpretations of midlife women’s sexual experiences and changes about sexual engagement, unwanted sexual experiences, body image, sexual healthcare.	High.	Increasing age and sexual-minority group associated with worse healthcare experiences. New lack of sexual desire: sometimes loss, sometimes relief. Relieved to no longer feel like sexual objects. After divorce-renewed sense of autonomy over sexual experiences, renewed interest in sex with new partners. Interdependence between their sexual experiences and other important people in their lives. Growth in sexual expression-increasing awareness of own sexual needs. Legacy of unwanted sexual experiences. Overwhelmingly negative healthcare experiences, felt dismissed, ageist, sexist or heterosexist attitudes – deep scepticism of HC system’s ability to meet needs. Representation matters – want more women and more minorities as providers. Those who also worked as HCPs expressed concern that there is very little training for HCPs about needs of midlife women. Positive experience if provider normalised age-related changes and provided honest and direct communication.
Weng et al. (2001), UK^131^	581, Aged 45–54, women, gender not specified, presumed cisgender	Quantitative. Cross-sectional study. Convenience sample.	Factors associated with physician recommendation of HRT.	High.	Black women significantly less likely than White women to report being advised about HRT.
Wigfall et al. (2011), USA^132^	2027, Aged 50–64, women, gender identity not specified, presumed cisgender, 6 deep-South States, majority had college or higher education level, majority Non-Hispanic White, but good Non-Hispanic Black representation	Quantitative. Cohort study. Convenience sample.	HIV testing uptake among postmenopausal women.	High.	26% Had ever had HIV test (excluding blood donation testing); 14% had most recent test during post-reproductive years. Women aged 50–54 were 25% as likely to have been tested for HIV as women 60–64. Non-Hispanic White women were half as likely as Non-Hispanic Black women, and rural women 30% as likely as urban women, to have had most recent test during post-reproductive years.
Wilson et al. (2013), USA^133^	10, Ages 28–55 years, women who identify as transwomen, African-American	Qualitative. In-depth interviews. Convenience sampling.	Barriers and facilitators to HIV care and support services.	Moderate(demographic details (age in particular) unclear, all participants same ethnicity).	Gender-related stigma: socially excluded and isolated; peer distrust; institutional distrust-negative experiences in HIV care system. Facilitators: instrumental support (incentives to meet their basic needs), emotional and informational support, access to gender-related care. Transportation and safe and anonymous location of services. Social connectedness.
Wong et al. (2018), Hong Kong^29^	40, Age 42–65 years, gender identity not specified, presumed cisgender, Chinese Cantonese	Qualitative. Interviews. Convenience sampling.	Impact of menopause on sexual health.	High.	Lack of information – suggestion to give information specifically about sexual health (how physical and emotional changes influence sex life and strategies) instead of general menopause symptoms. Cultural taboos – lack of open dialogue about sex and menopause – unsure what is ‘normal’. Seminars, pamphlets for women, documentaries, storytelling programmes, adverts, newspapers/magazines useful sources of information. Financial constraints to seeking healthcare.

CASP, Critical Appraisal Checklists; HCP, healthcare professionals; HPV, human papilloma virus; HRT, hormone replacement therapy; SHSW, sexual health and sexual well-being; STI, sexually transmitted infection.

### Study locations

Most of the studies were conducted in the United States (46), and the United Kingdom (17), with the remaining being conducted in Canada (7), Australia (2), Hong Kong (1), Israel (2), Spain (1), Italy (1), Ireland (1), Spain and Serbia (1), England, Finland, Denmark, New Zealand, Australia and United States (1) and Europe (1).

### Design of included studies

Studies included in the review were quantitative (45), qualitative (30) and mixed methods (six). Of those using qualitative methods, nine employed focus group discussions, 16 used interviews, four used both focus group discussions and interviews and one was a qualitative content analysis. Of those employing quantitative methods, one was a review analysis, 10 were cohort studies, 32 were cross-sectional studies and two were randomised trials. Mixed methods trials included cross-sectional studies linked with interviews (three) or interviews (one) and focus groups (one), a cohort study linked with interviews (one) and a mixed quantitative and qualitative content analysis (one).

### Study samples

Most studies (60) were presumed to have been conducted on cisgender women, although this was not specifically documented. Ten studies were conducted on transgender women. Three studies were conducted on both transgender and cisgender women. Eight studies were conducted on HCPs.

### Recruitment methods

Convenience sampling was the most common method of recruitment (51), followed by purposive sampling (20). Four studies used a mixture of purposive and snowballing sampling, one study used convenience and snowballing sampling, four studies used randomised sampling, one study used ‘mixed recruitment’.

### Quality assessment

The CASP checklists^57^ were used to critically appraise the studies with systematic consistency (Table 2). Studies were assessed for validity and methodological reliability. Sixty-five of the studies were deemed to be of high quality, 15 of moderate quality and one of poor quality.

### Type of SHSW areas examined

The enablers and barriers to accessing different SHSW services were examined. The SHSW services investigated included: sexual experience and function services, cervical screening, human papilloma virus (HPV) and cervical cancer care, HIV and STI testing, primary and preventive care, HIV care, HIV pre-exposure and post-exposure prophylaxis, menopause care, Hormone Replacement Therapy (HRT), gender-based violence services, contraceptive care and women’s health services in general. Thematic extraction of findings (Table 3).

**Table 3.** Thematic extraction of findings, based on the socio-ecological model. (specific to people who identify as trans and non-binary in italics)

Main themes	Sub themes: Barriers	Sub themes: Enablers
**Intra-personal** Intersecting disadvantage of marginalised groups Knowledge and awareness, and behaviourPsychological factor Competing priorities	Age. Sexuality. Physical appearance. Gender. Gender identity. Immigration status. Language. Cultural group. Racial and ethnic groups. Linguistic ability. Colonial legacy. Level of education. Income. Level of deprivation of area of residence. Area of residence (rurality). *Unstable housing.* Living with HIV. Sex work. Selling drugs. Previous incarceration. Accuracy, depth and breadth. Use inadequate indicators to assess STI status of partners, for example, demeanour, appearance. Difficulty in differentiating between credible and non-credible information. Internet deemed a confusing medium. Brochures and videos not specific enough and fail to address intensity of symptoms. Repetition of learned unhelpful behaviour, for example, only using condoms if worried about pregnancy. Learned fear: older women have seen women who left the community to get help not returning. Lack of familiarity with condoms. Lack of access to advice for younger women-older relatives are dead/cannot remember/will not talk about menopause. Depression. Loneliness. Poor self-esteem. Self-silencing. Low levels of sexual assertiveness. Felt defensive. Fear. Worry. Embarrassment. Less self-efficacy meant less likely to seek help from HCPs. Prioritising of intimacy over risk. Feelings of intimacy falsely associated with feelings of safety from STIs. Vulnerability of relationship transitions. New lack of desire felt as a relief. Sense of freedom linked with feeling young, resulting in more risk taking. Impact of traumaPhysical changes. Comorbidities (impact of physical and mental health and perceived health status). Stage of the menopause. Fear of side-effects and contra-indications to treatments. Concerned about effect of condoms on erectile dysfunction. *Prioritised gender-affirming therapy*. Lack of time. Care-giving responsibilities.	Age. Level of education. More likely to use condoms compared to when younger. Multi-format educational material, for example, consumer decision aids, seminars, pamphlets, documentaries, storytelling programmes, adverts, newspapers/magazines, comprehensive website. In-person options. Support groups. Need for specific information about sexual health during menopause. *Peer out-reach.* Making accessing services a habitual behaviour, for example, regular smear tests. Mobile health, for example, with podcasts, videos, virtual reality, mindfulness, expert blogs (anonymous, quick to access, evidence-based). No longer felt like sexual objects. Renewed sense of autonomy. Growth In sexual expression and awareness of own needs. Renewed interest in sex. New lack of desire sometimes felt as a loss (prompting help-seeking). Impact of trauma (prompting help-seeking). Stage of the menopause (severity of symptoms may prompt utilisation of other SHSW services).
**Intra-personal** Interactions with providers Women’s perceptions of HCPs HCP Knowledge and Beliefs Representation in healthcare	Made to feel dismissed. Ageist. Sexist. Heterosexist. *Transphobic.* Racist. Embarrassed. HCPs influenced by social taboos. More training required. HCPs reticent to initiate conversations. Uncertainty as to which HCPs could best answer their questions. Descriptions of physicians who make unilateral decisions. Need for better training. Prioritise general over sexual health. Do not ask about sexual well-being even if patients have conditions affecting sex life. Question whether menopause an issue for immigrant women. HCPs prefer women to enter consultations clear and informed about what they want. Women’s Initiative study.	Knowledgeable, approachable physicians. Supportive and trustworthy. Caring relationships. Holistic care. Personalised. Preventive care. Multi-skilled HCPs. HCPs who broach sexual health topics. HCPs normalise age-related changes. Honest and direct communication. Non-judgemental and do not make assumptions. Appetite for better training. Women should oversee decisions. Women who consulted HCPs found them useful. Decision aids could enable discussions More women. Better cultural and minority group representation.
**Organisation factors** Perception of HC systems Format of HC systems	Institutional distrust. Sceptical of system’s ability to meet needs.	Integrated educational and emotional support. Interdisciplinary clinics. Organisations incorporating many different services: One-stop, *Gender-affirming*. STI services incorporated into other healthcare service. Self-testing. Self-service. Women-only clinics. Multiple options of healthcare education. Culturally competent systems. *Anonymous locations*. Policy change to allow HCPs other than physicians to prescribe HRT. *Trans-positive hospital policies.*
**Community factors** Cultural factors Social connection Intergenerational factors	Stigma. Social norms (age and sex), for example age-gender barriers to accessing condoms in shops/pharmacies. Women view help-seeking appropriate only up to a certain age. Normalisation of condomless sex. ‘Othering’ those at risk of STIs. Paying for sex. Older people should ‘know better’-ashamed to seek help. Normalisation of violence. Mainly women’s responsibilities. Women socialised to minimise discomforts. ‘Couple culture’ – pressure to re-couple. Workplace expectations, minimisation by employees and colleagues. Reluctance to talk about sex with partner prevents help-seeking. Lack of understanding from partner. Sex less pleasurable due to partner or relationship characteristics, for example, partner’s inability to perform, unhappiness in relationship. Lack of recognition and awareness at work about menopause. Without awareness, women felt uncertain of the validity of their own experiences, often questioning themselves and their concerns. Distancing from young people’s risky behaviour yet pressure to adjust to new sexual culture fast. Ageist assumptions from younger men, for example, expectation that midlife women are lower risk so can have condomless sex. Fast progression to sex in new relationships. Condoms associated with youth. Mothers not discussing details of menopause with daughters.	Social norms: sex as a work-out, sex as beneficial to women’s health. Reconnecting with world. Educating others in community. Interdependence between their sexual experiences and important people in life. More likely to seek help if partnered. Gender-age dynamics on sexual risk negotiation: influence of young relatives on understanding of sexual safety. Fighting against stereotypes of older women not being sexually active.
**Public policy** Public health messages Material barriers/enablers	Disconnection from safe sex messaging. Sensationalised media coverage. Excluded by adverts. Cost of services. Transport options. Shortage of appropriate HCPs. Shortage of HCP time. Poor treatment options/lack of research.	Greater public discussion about sexuality and more positive mid-life representation. PrEP should be advertised all over the city, on the buses, metro stations (subways), on doors everywhere, in doctors’ offices, social media*-*everywhere possible, with phone numbers to contact, particularly on streets and public places because ‘I learn my information from the street’. Create identifying profile markers to improve focus of strategies. Focus on predictors of risk behaviour rather than predictors of risk status. Country-specific approach required. Facilities closer to home. Insurance coverage. Expertise and skills and range of tests available outside sexual health services. Recall-based screening systems. *Incentives (financial) to meet basic needs*. Mobile technology, for example, Apps.

The barriers and enablers to accessing sexual health and well-being services for midlife women (aged 40–65 years) in high-income countries: A mixed-methods systematic review. HC, healthcare; HCP, healthcare professionals; PrEP, pre-exposure prophylaxis; SHSW, sexual health and sexual well-being; STI, sexually transmitted infection.

## Intra-personal themes

### Intersecting disadvantage of marginalised groups

Twenty-eight studies revealed the intersecting disadvantage of being a midlife woman and belonging to other marginalised groups with relation to accessing SHSW services. There is evidence that age,^72,90,108,113,130,134^ sexuality,^108,130^ ‘physical appearance’ as a cause of perceived discrimination,^135^ gender,^90,134^ gender identity,^75,99,136^ race,^99,113,137^ ethnicity,^96,131,137^ immigration status,^61^ cultural group,^61,78^ nationality,^87,114^ colonial legacy,^98^ linguistic ability,^78,125^ level of deprivation,^89^ income,^79^ perceived income inadequacy,^121^ area of residence,^84^ level of education^79,82,84,138^ and belonging to a stigmatised group,^63,74,77,103,113^ impact on the ability of midlife women to access SHSW care. This intersecting marginalisation was demonstrated by an Australian study which investigated whether a ‘hub and spoke’ model would improve access to testing for STIs. Primary HCPs reported that they ‘rarely’ provided sexual health consultations for the most marginalised communities, which were identified as lesbian, gay, queer and transexual people, sex workers, people who inject drugs, certain ethnicities, the incarcerated, and refugees.^106^

Older age, within the midlife range, often inhibited access to SHSW services:^63,66,130,134,109^ increased age was associated with worse sexual healthcare experiences,^130^ being less likely to consider HIV pre- and post-exposure prophylaxis for HIV, older women who had experienced a premature menopause were less likely to receive treatment than their younger counterparts,^63^ and younger women were more likely to seek sexual health help following breast cancer than their older counterparts.^109^ Although older age inhibited access to some services, perhaps due to a combination of discrimination, self-advocacy and lack of current knowledge, the wisdom acquired with age, sometimes resulted in improved access to services. Confounding factors such as ethnicity, family poverty status, urbanisation and HIV exposure risk, may have affected the results, but a large cohort study in the United States demonstrated that women aged 60–64 were more likely to have been screened for HIV than women aged 50–54.^132^ Similarly, one small study, which investigated the sexual behaviour of midlife women who had experienced incarceration, suggested that (older) women were more likely to use precautions to protect against STIs than when they were younger, and were more likely to co-test with new partners pre-sex compared to when they were younger.^139^

Non-heterosexual women faced more barriers to accessing SHSW services than heterosexual women. They described hesitancy seeking help due to negative past experiences after disclosing their sexuality,^108^ and worse sexual healthcare experiences.^130^ Bisexual and homosexual women in the United States had lower cervical screening rates than heterosexual women;^140^ proposed reasons were difficult past experiences,^141^ and poor HCP knowledge about when screening was required.^142^ One study in the United States found that perceived discrimination due to ‘physical appearance’ (not defined), and gender, were associated with the reduced receipt of cervical smears;^94^ the authors suggested that the discrimination that these women perceived to be present may have inhibited their access to screening. The gender imbalance in sexual relationships remains an ongoing issue: two different UK studies found that both women and men frame the necessity to seek help for sexual difficulties predominantly in relation to erectile dysfunction.^8,90^ It can be postulated that this minimises the importance that women face on seeking help for their own sexual dysfunction. Gender identity also affects midlife women’s access to care:^99,103,115,136,143^ three studies demonstrated that transphobia was a strong barrier to enrolling in,^115,144^ and engaging in,^99^ HIV care. Transwomen also faced unique challenges when accessing care following sexual assault.^75^

Six studies demonstrated that race^99,113,132^ and ethnicity^61,131,137^ affected use of preventive healthcare,^60^ HIV-testing,^132^ HIV-related stigma,^113^ HIV care for transwomen,^99^ and awareness of HIV PrEP for transwomen.^137^ Similarly, Black women were significantly less likely than White women to report being offered HRT,^131^ and a study in Israel revealed that Arab women were more likely than Jewish women to present at an advanced stage of cervical cancer.^96^ Although, in contrast, one study found that perceived racial discrimination was not found to be associated with receipt of cervical smears,^94^ this research had many limitations including the unusually frequent exposure that the study participants had to healthcare services.

Access to HRT varied significantly depending on country of residence.^87,114^ Despite the fact that self-reported menopausal symptoms did not differ significantly among women of different nationalities, there was a marked difference in the utilisation of hormone therapy, for example with much higher use in France compared to Spain.^87^ An analogous finding was that linguistic ability was a barrier to accessing menopause care for Hispanic women in the United States,^78^ and affected women’s desire to participate in medical decision-making around menopause issues.^125^ Cultural inhibitions and the need for a higher level of assertiveness than previously required when dealing with American providers limited women’s access to menopause care,^78^ and an Australian study described the legacy of colonialism as a barrier to women accessing cervical smears.^98^

The social determinants of health,^145^ including income,^79,89,121,122^ area of residence,^84^ and level of education,^79,82,84,138^ have been associated with access to SHSW services for midlife women. Sexual and reproductive health behaviour was associated with socio-economic indicators in Spanish women who were born before the 1950s.^84^ Both socio-economic inequity in Canada,^98^ and lowest perceived income adequacy as an emotional barrier in the United States (higher fear of cancer being detected),^122^ have been associated with decreased access to cervical screening. Similarly, in the United States, higher level of income was associated with an increased likelihood of HRT counselling being obtained by women, although the results may be out-dated, as the study was conducted in 1998.^80^ A UK-based study found a strong association between HRT prescription rate and socio-economic deprivation. After adjusting for cardiovascular risks, there was an 18% lower HRT prescription rate in the most deprived practices compared with the least deprived.^89^ Poverty has also been cited as a major barrier to transgender midlife women accessing HIV care.^115^ Likewise, area of residence has been associated with access to SHSW services: American women living in urban areas were much more likely to have been screened for HIV than those living in rural areas.^132^ Level of education, a significant factor influencing access to public services, was found to impact on access to SHSW care.^61,79,82,98,134^ Five studies, investigating HRT,^82^ preventive healthcare use,^61^ STI testing,^134^ cervical screening^98^ and likelihood to receive HRT counselling,^80^ found positive associations between the level of a woman’s education and their ability to access SHSW services.

Belonging to a stigmatised group, such as being a sex worker,^144^ selling^144^ and using recreational drugs,^74^ having been recently imprisoned,^77^ and living with a condition such as HIV,^63,113^ negatively impacted on access to SHSW for midlife women. For example, engagement in sex work and selling drugs were identified as prohibiting factors for women who required HIV care in the United States.^103^ A Canadian study demonstrated that injecting drugs was associated with worse adherence to antiretroviral therapy for midlife women living with HIV.^74^ Recent incarceration was found to be independently associated with not achieving HIV virological suppression in a cohort study of women living with HIV in Canada.^77^ Although there are many reasons, apart from belonging to a minoritised group, why these women found accessing care challenging, establishing where care is most needed is the first step in disentangling where services should be focused.

### Knowledge, awareness and behaviour

Twenty-five studies exploring knowledge about STI risks,^24,70^ HPV,^120^ cervical cancer,^95,121^ menopause,^22,29,34,117,146^ HRT,^91,111,114,147^ contraception,^66^ HIV PrEP^62,65,85^ and HIV Treatment as Prevention,^129,148^ revealed a poor depth and breadth of SHSW knowledge among midlife women, which was a barrier to accessing services. The incorporation of different formats of education into healthcare systems^64,93,100,109^ was viewed as an enabler to accessing SHSW services.

Interviews with mid-lifers in the United Kingdom, following the end of long-term relationships, found that participants used indicators such as demeanour and appearance to assess the STI status of new partners.^24^ Similarly, very few heterosexual mid-lifers in Scotland used condoms or co-tested with new partners for STIs if their new partners reassured them that they were ‘low-risk’ (few previous partners, recently had a long previous relationship).^70^ Indeed, some women cited freedom from the risk of pregnancy as a reason not to use condoms, simultaneously (and perhaps unknowingly) increasing their exposure to STIs.^70,123^ Unfamiliarity, or inexperience, with condoms after not using them for a long time, also prevented their use.^24^ Women in the United States, as recently as 2018, demonstrated disappointing knowledge about contraception and emergency contraception^66^ and had poor knowledge about HPV, cervical cancer and HPV vaccination.^120^ In focus groups about HIV Pre-exposure Prophylaxis in the United States, women were surprised and angry that they had never heard about it; they advocated for educational campaigns.^85^ More reassuringly, although still in need of improvement, an Irish study found that 84% of women were aware of why they should have regular cervical smears.^95^ Reflecting the need for access to evolving health education at different stages of women’s lives, a study in the United States in the year 2000 found that women who reported never having had sex as adults had lower cervical screening rates and lower rates of HRT use.^140^

Middle aged women’s knowledge about menopause symptoms and management was disappointing.^22,34,73,81,126,146^ Focus groups in the United States reported a need for (more) reliable, accessible and current information about the menopause.^81^ In one study of women living with epilepsy, only 31% had confidence that they were knowledgeable about the menopause; the authors attributed this to inadequate patient–physician communication.^146^ An Italian survey found that a minority of midlife women received information about the menopause, and possible therapies, and those who did found it of poor quality.^73^ Of 3046 postmenopausal women with vulvar-vaginal atrophy, only 24% attributed their symptoms to the menopause.^34^ Breast cancer survivors with menopausal symptoms, in a study in 2013, described difficulty distinguishing between credible and non-credible sources of information, and uncertainty about which HCPs to approach for advice.^147^

The importance of multiple formats of SHSW educational resources for midlife women was highlighted,^22,64,93,109^ regarding sexual health following cancer treatment,^64,93^ sexual health concerns,^109^ the menopause^22,78^ and HRT.^100^ Women attributed the poor quality of information available about HRT as a barrier to making informed decisions, described the internet as confusing,^100^ and suggested a comprehensive HRT website, with regular seminars for menopausal women^100^ and consumer decision aids summarising the evidence about HRT.^100^ Hispanic women were disappointed that brochures and videos were non-specific and failed to address the intensity of their menopausal symptoms. They called for better tailored, individualised information and described a lack of access to advice due to the death of matriarchs, and cultural challenges with asking elders to educate them.^78^ Women in Hong Kong felt that information about the physical and emotional changes in the menopause which can affect sexual function should be specifically addressed by HCPs, and suggested seminars and pamphlets, documentaries, storytelling programmes, adverts, newspapers and magazines as useful sources of information.^29^ It must be acknowledged that historically limited research about the management of menopause compounded the lack of access to good information and may have influenced the findings of these studies.^149^ In a more recent study, nurses in the menopause transition described spending a long time looking for information, with poor results. Due to the widespread use of smart phones, they felt that it should be possible to have quick, easy access at any time in an anonymous way, secure platform with evidence-based information. They described the necessity for digital resources to be culturally sensitive, with podcasts, videos, virtual reality, mindfulness, and expert blogs.^22^ Survivors of breast cancer who requested education about sexual dysfunction had a preference for in-person options and support groups.^93^ Similarly, women (cisgender and transgender) who were consulted about how to improve their engagement with PrEP education advocated for learning from peers.^129^

A facilitator to accessing SHSW services was when women made visiting services into a habitual behaviour. Midlife women in the United States, where there was not a universal screening recall system, were significantly more likely to have a cervical smear if they had one previously.^121^ However, not re-assessing personal changes in risk with time and circumstance may have resulted in adverse outcomes for some women. One study found that women’s self-perceived STI risk was sometimes rooted in past long-term relationships: even if they were taking chances with their SHSW, they still viewed themselves as at the same exposure as when they were in a long-term monogamous relationship.^24^

### Psychological factors

Midlife is a particularly vulnerable phase for experiencing mental health problems.^150^ In the United States, rates of depression are highest in women in their midlife, compared to men, and women of other ages.^151^ Sexual health problems can be interlinked with mental health challenges,^152^ and poor mental health can significantly impact on a woman’s ability to access SHSW care.^70,99,102,121,122,124^ In addition, poor self-confidence has been associated with holding stigmatising beliefs,^135^ and risk-taking behaviour.^124^ Midlife women with low levels of self-esteem and sexual assertiveness, and high levels of self-silencing, were more likely to report HIV-stigmatising beliefs than women with higher levels of self-confidence.^135^ It can be postulated that holding such views prevented them from accessing services. Women in the United States cited depression, loneliness, low self-esteem and defensiveness, as reasons for engaging in high-risk sexual behaviours.^99,124^ Similarly in the United Kingdom, feelings of guilt and loss of self-esteem influenced sexual risk taking, and experiencing intimacy was prioritised, and seen as exclusive, to self-protection from STIs.^70^ Breast cancer survivors who had lower self-efficacy were more likely to seek help for sexual dysfunction from outlets other than HCPs.^109^ Discomfort with thinking about and discussing SHSW, can sometimes be attributed to culturally embedded psychological hurdles to accessing services. Fear, worry and embarrassment have been identified as barriers to cervical screening,^121^ two-thirds of postmenopausal women in one study were uncomfortable discussing vaginal atrophy,^102^ and discomfort with the topic of sexuality made it hard for cancer patients to seek psychosexual help.^153^ The impact of trauma can also pervade healthcare choices, for example one study demonstrated that the experience of trauma was a barrier for adherence to antiretroviral therapy for midlife women.^74^

However, some psychological factors, for example the sexual liberation often associated with ageing, have been shown to enable access to SHSW care.^123,130^ Older women living with HIV in the United States described many different elements which may have prompted them to seek SHSW services: aging was associated with sexual freedom, a growth in sexual expression and increased self-awareness.^123^ They described respite from feeling like sexual objects, articulated a renewed sense of autonomy over sexual experiences after divorce and conflicting feelings over a new lack of sexual desire.^130^

### Competing priorities

Midlife women have many competing priorities. Lack of time to look after themselves,^86,153^ care-giving responsibilities,^129^ increasing prevalence of comorbidities^8,61,92,123^ and worries about the side effects of treatment and interactions with other medications^60,87,90,115,146^ can present challenges to accessing SHSW services. Priorities also change with age and circumstance, for example as the risk of pregnancy diminishes,^70^ or the need for support with children decreases,^123^ factors that have had an impact on SHSW help-seeking, such as the balance of risks versus pleasure, may change.

Stage of the menopause journey was shown to be both a barrier and an enabler to accessing care.^74,105^ Women aged 40–50 years had better adherence to antiretrovirals than women aged less than 40; the authors suggested that in seeking help for menopausal symptoms, women were exposed to services more often.^105^ In contrast, symptoms could interfere with the ability to access care. Two studies found an association between severe menopausal symptoms and suboptimal antiretroviral adherence,^74,119^ and one study found an association between intensity of menopausal symptoms and reduced HIV clinic attendance.^119^

The fear of side effects from treatments (HRT,^60,87^ HIV PrEP^60,85^) worry about medication interactions (HIV PrEP on hormone use,^60^ hormones on antiretrovirals,^115^ antiepileptic drugs and HRT,^146^ comorbidity medications on sexual dysfunction treatment^90^) and other health issues sometimes took precedence over SHSW concerns.^76,129^ Women described the negative impact of comorbidities on their sex life, physical limitations in sexual activity, and changes in the experience of orgasm, factors which increased their need for, but complicated their access to, SHSW services.^123^ Poor self-rated health was associated with less use of preventive care.^61^ Women living with HIV were less likely to access hormone therapy for premature menopause than other women.^63^ In a UK study, the odds for being sexually active were lower for women who saw themselves as being in bad or very bad health compared with those in very good health.^8^ It can be postulated that poor health not only affects the ability to have and enjoy sex, but also to access SHSW services if more sexual activity or satisfaction is desired. Transwomen identified mental health problems as a significant barrier to accessing HIV care^63,115^ and prioritised gender affirming therapy over HIV PrEP.^99,115^ Midlife women in the United Kingdom sometimes spent years considering whether to access professional help for sexual difficulties,^90^ and 40% of women with vaginal atrophy in one study in the United States waited more than 1 year before consulting a HCP^117^; this could be attributed to many factors, including competing priorities for their time, attention and health.

## Interaction with providers

Four themes emerged about the barriers and enablers imposed by HCPs: women’s perceptions of the knowledge and beliefs of HCPs, the objective knowledge of HCPs, beliefs of HCPs about their own knowledge, and representation of midlife women in SHSW services.

### Women’s perceptions of HCPs

Negative encounters with HCPs, including being made to feel dismissed,^130^ not taken seriously^90^ and encountering ageist,^130^ sexist,^130^ heterosexist,^130^ transphobic,^99^ racist^99^ and HCP attitudes, fostered an ongoing distrust amongst midlife women of SHSW services. Women in one study in the United States, some of whom also worked as HCPs, felt that HCPs needed more training about the needs of midlife women.^130^ Women described poor access to competent physicians, as a barrier to accessing: sexual health following oncological treatment,^93^ sexual health in primary care,^76^ sexual health concerns,^104^ menopause care,^78^ sexual dysfunction services for postmenopausal women,^154^ transgender competent HIV PrEP care^65^ and transgender competent HIV care.^101^ In contrast, knowledgeable physicians were described by women as having enabled them to access HRT.^100^ The importance of multi-skilled HCPs who could deliver tailored care was highlighted by three studies: women described aspirations to receive personalised, preventative, truthful, unbiased menopause care^78,128^ and transwomen felt that HCPs should be competent in performing many different roles.^99^ Women felt that HCPs should provide more holistic care,^30,123^ for example they felt that physical activity should have been offered as a possible treatment for the menopause,^30^ and sexual satisfaction should have been discussed at the same time that STI care had been offered.^123^ Women felt that HCP’s prioritisation of general health over sexual health^34^ was a barrier to SHSW services.

Behaviour, such as communication skills,^128^ displayed by HCPs influenced midlife women’s access to SHSW care. Women wanted to feel equal in the decision making relationship,^81^ and physicians who made unilateral decisions^100^ were viewed as barriers to care. Approachable HCPs, who were non-judgemental and did not make assumptions about them,^108^ and who were able to develop a good rapport,^76^ and normalise age-related changes,^130^ encouraged them to access services. Physicians across primary and secondary care and a range of specialties were reticent to initiate conversations about sexual health,^76,90,154^ yet women stated that they would have preferred physicians to broach sexual health topics.^55,102,105,108,124,150^ Women surmised that HCPs did not broach sexual health topics due to embarrassment, ageism and social taboos around older women and sex^76^; it was not specified whether the gender of the HCP made any difference to women’s assumptions.

### HCP’s knowledge and beliefs

A range of HCPs support the SHSW of midlife women; the review found there was a need for better training in this subject across this group of professionals.^69,75,90,106,112,146^ In one study, 52% of women living with epilepsy who had discussed the menopause with their HCP still wanted more information.^146^ In the United Kingdom, there were too many missed opportunities to test women for HIV, which had resulted in (preventable) irreversible consequences.^112^ Similarly, a different study found that contraception had only been documented to have been offered to 15% of eligible women living with HIV.^69^ HCPs questioned whether menopause was even an issue for immigrant Hispanic women, indicated that socialisation to minimise gynaecological discomforts limited the significance of their symptoms, and believed (without any evidence) that women were managing their symptoms through local medicine women.^78^ The majority of women in one study, despite having conditions which were known to affect sexual function, had never been asked by their GP about sexual well-being.^90^ Furthermore, a large survey in the United States which explored sexual concerns, found that most women wanted to discuss these with a HCP (but had not managed to), but encouragingly the discussion had proved helpful for those who had done so.^104^

Compelling evidence demonstrated an appetite among HCPs for better, wider-ranging training.^67,75,83,88,106,110,127^ A questionnaire for community HCPs about HRT,^110^ a questionnaire eliciting nephrologists’ confidence in SHSW for women,^88^ a questionnaire investigating barriers to sexual dysfunction screening,^127^ interviews exploring competence in sexual healthcare,^67^ a questionnaire which determined HCP trainees’ views in delivering perimenopausal care,^83^ a study of HCPs caring for survivors of sexual assault^75^ and an evaluation of a hub-spoke model,^106^ verified lack of HCP confidence in SHSW issues, and a desire for further education among HCPs. Training needs identified included taking a non-judgemental sexual history, using culturally appropriate terms, and management of work flow in the clinic.^106^ The initial findings of the Women’s Health Initiative, a large-scale national trial, influenced the professional and public health narrative about the dangers of HRT,^64^ and thereby controversially continues to negatively impact on practitioner’s HRT prescribing rates.^155^ However, a positive fall-out of the study was that many HCPs expressed the feeling that patients should be the main decision-maker with regard to HRT.^64^

### Representation in healthcare

A UK-based study which explored women’s midlife transitions,^130^ and a study in the United States which investigated unmarried midlife women’s sexual health-seeking behaviour,^108^ found that midlife women welcomed more female representation in healthcare. Women in the United States expressed a preference for an older female provider when discussing menopause treatment.^71^ Correspondingly, women with type 2 diabetes viewed female HCPs as facilitators to them accessing sexual healthcare discussions,^76^ and having a female physician correlated with women having had a cervical smear.^86^ Arab women’s low use of preventative healthcare was related to a lack of physicians of the same culture and gender.^61^

## Organisational factors

### Perceptions of healthcare systems

Institutional distrust was a barrier to healthcare services^130^ within the remits of: access to HRT,^100^ HIV care for women,^113^ and HIV care for transwomen.^115,123,133,143^ Systems which offered emotional and information support together with clinical care were viewed as enablers to accessing care for transwomen.^133,156^

### Format of healthcare systems

Strategies to make healthcare systems more accessible that were proposed centred around combining expertise^88^ and services,^106,133^ and establishing women-only clinics.^54,123^ Nephrologists suggested that interdisciplinary clinics could improve their ability to manage women’s SHSW needs.^88^ Similarly, combining SHSW services,^53,54,99,101,129,133,135^ co-locating SHSW services within other existing healthcare services,^99^ and self-service and self-testing possibilities,^97,122^ were suggested as possibilities to improve access. HCPs in one study felt that a recall-based system, employed in many other countries, would have improved cervical screening rates among an Aboriginal community in Canada.^98^ Canadian women in this study also advocated policy change to allow health professionals other than physicians to prescribe HRT.^100^ This would improve access, by increasing the availability of services. Enablers for access to SHSW services that were suggested for transwomen included creating trans-positive environments and services with trans-positive policies,^75^ co-location of gender-affirming and other SHSW services,^54,99,101,133^ and including social care provision.^129^ Transwomen preferred services in a safe space away from men,^54,123^ and valued anonymous services.^133^

## Community factors

### Cultural factors

Although stigma is a cross-cutting theme which affects all levels of the SEM,^157^ here we concentrate on the effect of stigma in the community as a barrier to SHSW care. Fourteen studies attributed community-based stigma to be a barrier to midlife women accessing a wide range of SHSW care: sexual function care,^90^ postmenopausal sexual healthcare,^154^ menopause care,^22,29,78^ HIV care,^113^ preventive sexual health services^135^ and specific to transwomen: sexual health,^54,133^ HIV PrEP^60^ and HIV care.^99,103,115,133^

Social norms influenced SHSW care-seeking behaviour. For example, normalisation of condomless sex with new partners, paying for sex and ‘othering’ of those at risk of STIs, were barriers to SHSW services.^24^ Women experienced age-gender barriers to accessing condoms in shops and pharmacies^24^ and described seeking help for sexual difficulties as only being appropriate up to a certain age.^90^ Cultural expectations of stoicism around menstruation and menopause prevented Hispanic women from seeking help.^78^ Similarly, it can be postulated that the stigma and lack of understanding from healthcare colleagues prevented nurses in the menopause transition in one study from seeking help. They described how ‘tiredness related to menopause symptoms was not socially acceptable’, and one woman described how (at work) ‘you just get on with it, that sort of thing’.^22^

Evolving cultural landscapes may have complicated women’s ability to access SHSW services. A study which investigated gender-based violence described how the (increasing) normalisation of violence affected the healing process,^118^ and this cultural norm may have been a significant barrier to accessing care for some women. At the conclusion of a long-term relationship, some women felt pressured to re-partner to fit into (current) ‘couple culture’,^24^ whereas others described how sexual liberation from the fear of pregnancy, and from the traditional gender norms of when they were younger, such as the expectations of being in a committed relationship and needing financial support,^123^ meant that they had chosen more partners and sex whenever they wanted, compared to in their younger years.^123^ These changes in cultural expectations may have contributed to midlife women struggling to identify when to use SHSW services. However, prevalent social ideas may have prompted some women to seek SHSW care. For example, a content analysis of women’s magazines in the United States found the portrayal of sex as good exercise which benefitted the health of women, and the implication that a woman was responsible for her husband’s sexual fulfilment, as recurring themes,^68^ both of which may have encouraged women to access SHSW services.

### Social connection

Relationship dynamics and communication affected whether safe sex was practised.^123^ Women who were partnered were more likely to seek help for sexual health concerns after breast cancer treatment than those without a partner,^109^ but partnership with somebody not interested in (or capable of) sex significantly contributed to sexual inactivity.^8,92^ One woman described ‘missing the closeness of cuddles before and after’ but also ‘feeling sorry for my husband’ (beta-blockers and arthritis affected his ability to have sex).^8^ Another woman explained that because the sex practices she had previously enjoyed with her partner were no longer possible, they did not want to explore alternatives.^8^ In a different study, women described sex as less pleasurable compared with when they were younger due to their partner’s impaired sexual performance, and unhappiness and the feeling of being trapped in sexless relationships.^123^ These relationship complexities may have made accessing help challenging, even though it may have been desired. Conversely, women in a UK study described continuing to have sex despite a health-related reduction in their own sexual desire for the benefit of their partner.^8^ This highlights the need for holistic SHSW services for older couples, which include relationship counselling. Anxiety about communicating with intimate partners had a negative impact on access to services. One study demonstrated how inhibitions relating to conversations about sex with their partner prevented older adults from seeing professional help for sexual difficulties.^90^

Studies which investigated the nature of healing from gender-based violence,^118^ the repercussions of trauma experienced by transwomen living with HIV,^54^ and the legacy of unwanted sexual experiences on midlife women,^123^ highlighted themes which encompassed the need to learn how to reconnect with others, and the importance of belonging to a community in terms of facilitating access to SHSW services. Similarly, an exploration of the inclusion of transwomen in HIV services found that ciswomen’s openness to education about trans issues was much better than the trans community expected. If their receptivity had been recognised, social connection and enhanced inclusivity in services may have been accomplished more successfully.^54^ Without awareness and understanding from colleagues, nurses in the menopause transition felt judged and uncertain of the validity of their own experiences.^22^ It is likely that if women working in healthcare settings are sometimes expected to minimise their menopause symptoms, women in other workplaces may also be discouraged from accessing care by the expectations of employers and other employees.

### Intergenerational factors

Midlife women matured in a context of different sexual norms, sex education and SHSW campaigns. Midlife nurses in one study explained how female relatives had not helped to prepare them for the menopause by not discussing their experiences in any detail or depth.^22^ In contrast, one study demonstrated that younger relatives positively influenced midlife women’s understanding of sexual safety, and thereby enabled access to care.^24^ Women in a study in the United States railed against ageist stereotypes about their sex lives. They narrated how younger generations thought older women should not be sexually active, and how younger men perceived sex with older women as lower risk behaviour, and therefore expected condomless sex.^123^ Their ability to challenge this fallacy may have empowered them to access appropriate SHSW services. In contrast, other studies depicted midlife women as othering themselves from young people’s behaviour, despite engaging in similar risks. While midlife women perceived condoms as associated with youth,^24^ they also described pressure to adjust to a new sexual culture quickly, leading to rapid progression to sex in new relationships.^70^

## Public policy

### Public health messages

In a study about risk taking in 40–65 year olds, participants described disconnection from safe sex promotional services.^24^ The study identified a discrepancy between the positive broader public discussion about sexuality, and the sensationalised media coverage of sex in older women.^24^ These confusing messages may have contributed to increased stigma and discomfort around sex in the midlife and may have affected women’s decisions to access SHSW services. When discussing HIV PrEP campaigns, there was a concern that information was not available in the places that midlife women would frequent: they advocated for it to be advertised all over the city on the buses, metro stations, on doors, in doctors’ offices and social media, with phone numbers to contact.^85^ Transwomen described the need for better quality of public health campaigns. They worried about the need for clarity in messages about the lack of interactions between feminising hormones and HIV PrEP^65^ and felt that HIV PrEP adverts needed to include trans-specific messages^60^ in order to begin to tackle some of the barriers to uptake.

Although there are many barriers and enablers to SHSW care that are uniform across high-income countries, public health messages may benefit from being country specific. An international survey of four high-income countries found marked differences between the nations: 51% of postmenopausal women in the United States compared to 10% in Finland were aware of local treatment options for vaginal atrophy.^158^ There was a similar significant discrepancy between high-income nations in a study which examined HRT prescription rates.^87^ The identification of risk behaviours and group or nation-specific problems could help to target messages, for example, Parkes’s latent class analysis of NATSAL-3, which identified six classes for women, including ‘unwary STI risk takers’.^107^

### Material barriers

The material barriers to accessing SHSW which were identified included: financial challenges,^121,133^ transport difficulties,^121,129^ shortage of both HCPs and the time that they can offer^78,83,98,100,127,128^ and poor mainstream treatment options.^78^ Cost of care was found to be a barrier to accessing cervical screening^122^ and HIV care^133^ in the United States, SHSW services during the menopause in Hong Kong,^29^ and bioidentical HRT in Canada.^100^ Transwomen living with HIV proposed that financial incentives to meet their basic needs would be a facilitator to accessing HIV care.^133^ Similarly, some women discussing HIV PrEP in focus groups in the United States felt that financial incentives should be attached, although others felt that preventing HIV itself should be the incentive.^85^ Limited transport options were found to be barriers to accessing cervical screening in Canada^98^ and the United States,^98,122^ and HIV care in the United States.^133^ Both women and HCPs felt that better availability of HCPs would improve access to SHSW services. A shortage of appropriate HCPs^98^ and not having a primary care physician^86^ were associated with worse cervical smear uptake in the United States. HCPs felt that lack of consultation time was a significant barrier to providing good SHSW services,^78,83,127^ particularly in relation to menopause care^78,83^ and sexual dysfunction care.^127^ The lack of treatment options available for conditions affecting midlife women’s SHSW, such as sexual dysfunction,^90^ and menopause,^34,87^ may inhibit women from seeking, and HCP from offering, some SHSW services. A woman in one study expressed ‘I talk to mums everyday about babies and everything . . . there are so many variations on how it is for premenopausal women, like maybe we don’t really understand enough’.^22^ The lack of prioritisation for research into treatment options for conditions that affect midlife women means that even for those women who are able to access care, it can often feel fruitless.

Mobile technology, for example, applications have been used to improve access to menopause services.^116^ An analysis of 18 menopause applications found that data reports and visualisations encouraged application users to seek out help and facilitated conversations with HCPs.^116^ In particular, applications with clear links to HCP support were viewed positively by application reviewers, and all of the three applications with HCP support had ‘good’ quality scores.^116^ Importantly, however, few of the menopause applications were explicitly supported by HCPs, and many scored poorly in relation to the credibility of the source.^116^ A different study corroborated this: only 22.7% of the applications analysed had documented evidence-based practice in the form of guidelines or treatment protocols.^41^

## Discussion

To our knowledge, this is the first systematic review of the evidence that addresses the barriers and enablers to SHSW care for women aged 40–65 years in high-income countries. The gender disparity in healthcare research,^159^ the ethical challenges of conducting SHSW studies^160^ and the sociocultural pressures of the midlife phase,^18^ together with the increasing prevalence of general health issues which may both impact on sexual health and facilitate increased access to HCPs,^24^ resulted in limited, evolving and sometimes conflicted evidence with regard to the factors affecting access to services. Most research to date has identified the barriers and enablers that cisgender women encounter in accessing sexual health services. Encouragingly, there is increasing representation of transwomen in the literature and a developing recognition of the importance of sexual well-being.

The limited number of interventions that have been trialled to improve access to SHSW services for midlife women have mostly been found to be efficacious.^161^ The majority of successful interventions have included education or knowledge sharing (including health literacy and support and healthcare system navigation), been tailored to the specific population or sub-population (e.g. cancer survivors) and have included a diversity of methods of communication with which to engage participants.^162–168^ Studies indirectly exploring interventions to improve access to SHSW care in midlife women living with and surviving cancer provide valuable insights to guide future research. Examples include evidence for readily accessible self-treatments such as comparison between vaginal lubricants in breast cancer,^169^ physical activity to improve sexual well-being in endometrial cancer^170^ and technological psychoeducational interventions for breast, colorectal and gynaecological cancer survivors (telephone, text and internet approaches) which were found to reduce geographical barriers and confer convenience (ill health, competing care giving/work responsibilities) and privacy advantages.^166,171^

Consistent evidence has emerged about the wide disparity in access to care within the high-income setting. The intersecting disadvantage of being a midlife woman from an under-resourced group provides a clear indication as to where services should be focused. There is compelling evidence, and appetite for, multi-format, updated, education about SHSW issues and services for midlife women, their communities and their HCPs (across specialties^166,172^), not least so that improved, more equitable care can be advocated for. Mobile technology has been employed to democratise menopause education, but information on applications must be evidence-based^40^ and extended to other SHSW issues. We must capitalise on both intergenerational and peer-led learning and targeted community action^173^ to address unacceptable norms such as the socialisation of women to minimise discomfort, the framing of sexual dysfunction from a solely male perspective and to destigmatise and improve inclusivity of sexual health and well-being services for all midlife women.^167,174^ Public health messages focusing on predictors of risk behaviour, offering more evidence-based self-care interventions^175–177^ and steering the cultural discourse to positive, safe, enjoyable midlife sex for women are required. The impact of poor mental health provision on access to SHSW services should not be underestimated.^162,165,178^ The false economy of denying midlife women the holistic, free, easily accessible and integrated SHSW services that they deserve must be recognised.

### Strengths and limitations of the systematic review

The variety of study designs, comprising of qualitative, quantitative and mixed methods studies, prevented meta-analysis of findings. However, the inclusion of a wide range of methodologically diverse studies, and the narrative synthesis, enabled the emergence of complex themes, providing an in-depth understanding of the barriers and enablers to SHSW care for midlife women. The qualitative evidence complemented the quantitative results. For example, cohort and cross-sectional studies identified the more marginalised groups of women, and qualitative findings helped to describe the barriers faced by those women in their own words or the words of their HCPs.

SHSW services are deliberately combined in this review in an effort to support the World Health Organization’s approach of acknowledging positive sexuality and sexual experiences as key public health outcomes that are intrinsically linked with sexual health.^11^ However, SHSW services are inconsistently conceptualised. Although the review sought to include all SHSW services as per Mitchell’s 2021 framework,^11^ SHSW services in high-income countries that have been defined in different ways may not have been captured. Many of the included studies excluded an important group of transgender and gender non-binary people who have SHSW needs and experiences that are similar in some ways, but also unique, to those of cisgender women.^179^ Research lags behind our understanding of the imperative necessity to recognise and distinguish between the needs of women with non-binary and transgender identities. Most studies included in the review are presumed to have been conducted on cisgender women, but we are unable to differentiate women who were not asked, and those who did not want to reveal their gender identities. Therefore, we are unable to judge whether the review reflects access to care for non-binary and transwomen.

The exclusion of non-English language articles may have resulted in the lack of representation from important national and cultural perspectives. Furthermore, most studies were conducted in English-speaking countries, which impacts on the generalisability of the results for women living in non-English speaking countries. Although this review investigated the barriers and enablers to accessing care for women in high-income countries, only a small number of studies specifically addressed access to care for women resident, but who had not been born in, these countries. These women have unique needs, and further research is required to elucidate how to distinguish their challenges in accessing care from women resident in the countries in the high-income countries in which they were born.

### Strengths and limitations of the included studies

Overall, the quality of the reporting of the studies was high, with sound methodology and valid results. Studies addressed clearly focused issues. Study methodologies were appropriate for the aims of the studies, and closely aligned with the outcome measures. Half of the studies did not include the race or ethnicity of the participants, which may have had a significant bearing on the barriers and enablers to accessing care. When race or ethnicity was specified, most studies attempted to include women of different races and ethnicities, with mixed results.

Strong theoretical foundations for the work were clearly elaborated in most studies. Many studies used convenience sampling strategies, which may have resulted in an overrepresentation of women who were better able to overcome barriers to accessing care, and women who were already seeking care. Qualitative studies used focus groups, interviews or a mixture of both methods considered appropriate for eliciting views on sensitive topics such as SHSW.^180^ However, few of the qualitative studies reported on the quality assessment criteria of ‘reflexivity’, the acknowledgement of cultural bias in the researcher–participant relationship. Many of the quantitative studies were cross-sectional studies, either cross-sectional designs or cross-sections from other research samples. Most surveys were administered individually and verbally, modes considered most accessible for those with low literacy.^180^ However, some surveys required computer literacy, which will have excluded more marginalised and women in the older bracket of midlife, rendering the findings less generalisable.

### Implications for clinical practice and future research

#### Clinical practice

SHSW is interlinked with many aspects of mental and physical health and should therefore be provided as an important component of holistic care. Midlife can be viewed as a time of opportunity, where education about positive links between the adoption of sexual health and well-being behaviours can promote a healthier older age. HCPs with a range of roles and responsibilities, require and desire better training in delivering, and signposting, midlife women to SHSW services, and should therefore be offered appropriate training to give them the confidence and ability to do so in an inclusive, sensitive manner.

#### Policy

Maintaining an active, satisfying and fulfilling sexual life as women age is positively linked with life satisfaction.^182^ It is evident that policies which optimise SHSW as integral parts of holistic healthcare benefit women and the societies in which they live. There is a crucial need to advocate for policy change and social campaigns aimed at raising awareness among policymakers, workplaces and communities about the SHSW needs of midlife women. Such initiatives are essential for ensuring that appropriate resources and support are allocated to address the unique challenges faced by this demographic. This review demonstrates that midlife women can enjoy the changes that midlife brings to their SHSW but that they face many barriers to accessing research and services. Policies to educate the public and de-stigmatise SHSW and SHSW services are overdue. Women, especially those from the most under-served groups, must have access to high-quality information; this will help to address other barriers to care such as gender-age dynamics and harmful social norms. The competing priorities that midlife women juggle must be acknowledged within policies, by ensuring flexible appointment times, childcare options, knowledgeable employers and considering accessible one-stop or integrated and interdisciplinary services, with innovative delivery such as self-service and self-testing. Inadequate consultation time and lack of skilled HCPs are pervasive issues, and in countries where women pay for SHSW services, cost is a barrier to vital services.

#### Future research

Research about how to best enable the participation of midlife women, particularly those who belong to under-resourced groups, in SHSW research is needed. Midlife women’s voices can then be used to prioritise management strategies, including treatment options, education, use of technology and implementation of accessible services. Future research should include longitudinal cohort studies for in-depth examination of the sexual health and well-being challenges of midlife women, randomised controlled trials, with implementation science elements, to test service designs, for example women-only services, and targeted public health messages, as well as qualitative research such as in-depth interviews, focus group discussions and co-creation of education and policies, to ensure that midlife women’s voices are always at the forefront of new policies.

## Conclusion

The advent of the physiological and psychological changes that midlife brings to women can serve as an opportunity for workplaces, HCPs, communities, healthcare organisations and women themselves to evaluate, improve access to and co-create essential sexual health and well-being services. Implementation of changes must be evidence-based, and address the wide disparities in access, and the intersecting disadvantages of under-served groups.

## Supplemental Material

sj-docx-1-whe-10.1177_17455057241277723 – Supplemental material for The barriers and enablers to accessing sexual health and sexual well-being services for midlife women (aged 40–65 years) in high-income countries: A mixed-methods systematic review Supplemental material, sj-docx-1-whe-10.1177_17455057241277723 for The barriers and enablers to accessing sexual health and sexual well-being services for midlife women (aged 40–65 years) in high-income countries: A mixed-methods systematic review by Kiersten Simmons, Carrie Llewellyn, Stephen Bremner, Yvonne Gilleece, Claire Norcross and Collins Iwuji in Women’s Health

## Declarations

**Ethics approval and consent to participate:** Not applicable.

**Consent for publication:** Not applicable.

**Third-party submission:** Not applicable.

**Writing assistance:** Not applicable.

**Availability of data and materials:** Not applicable.
